# Precision-based exercise protocols for children with cancer: a methodological approach from the European FORTEe research project

**DOI:** 10.3389/fped.2026.1907775

**Published:** 2026-09-17

**Authors:** Francesca Lanfranconi, Elias Dreismickenbecker, William Zardo, Tommaso Moriggi, Emanuele Villa, Giorgia Radaelli, Eleonora Corti, Linda Peli, Marie A. Neu, Mareike Kuehn, Lena Wypyrsczyk, Carmen Fiuza-Luces, Alejandro Lucia, Miriam Götte, Martin Kaj Fridh, Lidija Kitanovski, Barbara Konda, Hayley Marriott, Rodolf Mongondry, Filippo Spreafico, Joachim Wiskemann, Adriana Balduzzi, Joerg Faber

**Affiliations:** 1 Centro Maria Letizia Verga - Fondazione Monza e Brianza per il Bambino e la sua Mamma, Monza, Italy; 2 University Medical Center of the Johannes Gutenberg-University Mainz, Childhood Cancer Center Mainz, Mainz, Germany; 3 Fondazione IRCCS Istituto Nazionale dei Tumori, Pediatric Oncology Unit, Milano, Italy; 4 Hospital 12 de Octubre Research Institute, ‘imas12‚, Physical Activity and Health Laboratory, Madrid, Spain; 5 Department of Sports Sciences, Faculty of Medicine, Health and Sports, Universidad Europea de Madrid, Madrid, Spain; 6 University Hospital Essen, West German Cancer Center, Essen, Germany; 7 University Hospital Copenhagen, Rigshospitalet, Department of Pediatrics and Adolescent Medicine, Copenhagen, Denmark; 8 University Medical Center Ljubljana, Division of Pediatrics, Department of Haematooncology, Ljubljana, Slovenia; 9 Forma 3D Ltd., Forma 3D, Ljubljana, Slovenia; 10 School of Sport, Nutrition and Allied Health Professions, Faculty of Health Science, and Technology, Oxford Brookes University, Oxford, United Kingdom; 11 Centre de Lutte Contre le Cancer Léon Bérard, Prevention Cancer Environment Department, Lyon, France; 12 Oncology Unit, IRCCS Istituto Giannina Gaslini, Genova, Italy; 13 Department of Medical Oncology, Working Group Exercise Oncology, a partnership between DKFZ and University Medical Center Heidelberg, Heidelberg University Hospital and National Center for Tumor Diseases, Heidelberg, Germany; 14 Pediatric Department, Fondazione IRCCS San Gerardo dei Tintori and School of Medicine and Surgery, Milano-Bicocca University, Monza, Italy

**Keywords:** adolescents, cancer, children, exercise, protocol

## Abstract

**Introduction:**

Children, adolescents and young adults with cancer face severely reduced exercise tolerance while undergoing treatment, which places an additional burden on their health. The FORTEe research project, a European multicenter randomized clinical trial, investigates if a precision-based exercise program (PEx) can reduce cancer-related fatigue and maintain or improve a broad range of physical and psychosocial outcomes, thereby supporting its integration into standard paediatric oncology care from the start of cancer treatment. This methodological paper describes how the protocols were developed, applied in the field, and disseminated within the scientific community.

**Methods:**

One year of consultation between the 10 clinical centers across the FORTEe consortium in Europe lead to the FORTEe precision-based exercise protocols (FORTEe-exprot). They include cardiorespiratory, resistance, flexibility, balance, and coordination training, tailored to age, functional status, and treatment intensity. Sessions are intended to be delivered 3–5 times/week, 20–60 minutes, in inpatient, outpatient, and home settings, with in-person or telehealth supervision. Safety considerations comprised individualized modifications, vital signs monitoring, and structured reporting of adverse events. Daily documentation of exercise type, intensity, duration, and individual perceived exertion informs ongoing prescription adjustments.

**Results:**

A final version of the FORTEe-exprot was ready by March 2022, prior to the start of clinical trial recruitment. During their development, potential risks were carefully considered, including injury, staff experience, data quality, recruitment, and compliance. The FORTEe-exprot were designed to be inclusive, accommodating children with varying needs and ensuring applicability across different levels of expertise among exercise professionals and diverse clinical settings. The FORTEe-exprot printed handbooks were disseminated internally and externally via conferences and distribution of around 50 countries worldwide. They are freely downloadable from the FORTEe website and from Zenodo, an open research repository commonly used for EU-funded research projects.

**Discussion:**

Effective implementation of PEx requires appropriately trained exercise professionals, risk-stratified prescription, daily documentation, and close interdisciplinary collaboration to ensure safe, individualized interventions aligned with each child's clinical status. Such multi-stakeholder collaboration constituted a key foundation of the FORTEe-exprot, highlighting their potential role in pediatric oncology care and their possible contribution to future evidence-based recommendations.

## Introduction

Children, adolescents and young adults with cancer (CAYA-c) face severely reduced exercise tolerance while undergoing cancer treatment, which places an additional burden on their health (1–13), which may persist also after the end of cancer treatment, ultimately limiting individuals' overall development and quality of life (14–18).

Of note, there are currently an estimated 500,000 childhood cancer survivors in Europe who may be facing late effects of cancer treatment, i.e., chronic diseases affecting their exercise tolerance and, mostly, their quality of life (19–21). Two-thirds of long term CAYA-c survivors will have late effects of treatment, which impact their daily life (22–24). International childhood cancer societies are committed to raising awareness among childhood cancer survivors to take responsibility for their own care and to encourage stakeholders and national health systems to improve their long-term follow-ups (25). Within this context, the early integration of precision-based exercise (PEx), initiated shortly after diagnosis, represents a valuable therapeutic investment aimed at preserving long-term health and mitigating the cumulative burden of late effects (26). Framing PEx as an integral component of cancer care from the outset, rather than a delayed rehabilitative strategy, positions exercise as a protective resource for future survivorship.

To date, only a limited number of randomized controlled trials have investigated exercise interventions in pediatric oncology during active cancer treatment (26–30). Furthermore, the generalizability of existing findings is hindered by small sample sizes and heterogeneity in study designs. To address these limitations and establish a robust evidence base, a multinational, multicenter European randomized controlled trial FORTEe was initiated in 2021, and the research project is scheduled for completion in August 2026. The FORTEe trial evaluates the effects of PEx in CAYA-c undergoing cancer treatment, with the contribution of experts from eight European countries, in a collaborative effort to develop standardized PEx and assessment protocols, implemented with the support of digital technologies (31).

An extended description of the FORTEe research project rationale and design has been previously reported by Neu et al. (31). Over the past five years, the FORTEe research project has generated considerable interest among exercise professionals, rehabilitation teams, oncologists, and parent associations worldwide. This growing attention stems from the broad dissemination of the project's objectives through multiple channels, including scientific conferences and the emergence of thematic working groups (e.g., the SIOP Rehabilitation and Physical Medicine working group, the SIOPE Exercise Oncology working group and the ECSS Exercise Oncology special interest group) within major pediatric oncology–related clinical and exercise and sport sciences organizations.

This methodological paper describes how the FORTEe precision-based exercise protocols (FORTEe-exprot) were developed, applied in the field, and disseminated within the scientific community. It examines the key factors supporting their delivery, including stakeholder engagement, to provide safe and effective training for CAYA-c. Furthermore, we present a standardized and pragmatic methodology developed within the FORTEe trial to harmonize exercise prescription across clinical centers with varying levels of experience among exercise professionals and different available facilities. The methodology also considered the inclusion of the most frail children, ensuring that the FORTEe-exprot could be adapted to accommodate a wide range of clinical conditions and functional capacities.

### FORTEe precision-based exercise protocols in CAYA-C

The FORTEe-exprot are freely downloadable from the FORTEe website and from Zenodo, an open research repository commonly used for EU-funded research projects and are distributed under a Creative Commons (CC) license. Their rationale is based on the premise that implementing PEx in CAYA-c requires a cultural shift in oncology care, whereby exercise is integrated as a core component of treatment from the first weeks after diagnosis to prevent deleterious effects on skeletal muscle, cardiorespiratory fitness, and bone health (13, 27, 32–37). Its benefits might extend beyond a simple improvement in exercise capacity and include improved feelings of physical self-perception and satisfaction with life (30, 38–41). PEx works on intensity, volume, frequency, and recovery, and optimizes individual physiological and psychological adaptations (32, 42–44).

There is a clear need for evidence-based exercise prescriptions to maximize both the performance-enhancing and therapeutic benefits of exercise. Emerging evidence positions exercise as a clinically meaningful adjunct therapy in oncology, with potential benefits on disease-free and overall survival too. Landmark trials, such as the CHALLENGE study in adults with colon cancer, demonstrate that structured aerobic exercise can yield survival benefits comparable in magnitude to established cancer therapies while enhancing quality of life (45). Translating these findings to CAYA-c, however, requires careful consideration of developmental stage, treatment-related toxicities, and long-term sequelae. In this context, the prescription of PEx must be individualized, and safely delivered, with particular attention to appropriate dosing, timing, and modality across the treatment strategy. Access to qualified exercise professionals and suitable clinical or community-based facilities is essential to maximize therapeutic benefit while minimizing risk.

Also, consistent with ACSM and ESSA recommendations, the systematic adjustment of training variables, such as the intensity of cardiorespiratory and resistance exercises, guided by both objective measures and subjective ratings of perceived exertion, is essential to ensure safety and optimize therapeutic outcomes (46). Failure to individualize programs may limit the full therapeutic potential of exercise, highlighting the necessity of tailored approaches that account for each patient's unique clinical status, functional capacity, and treatment-related constraints.

### Eligibility and intervention

Participants aged 4 to 21 years with a diagnosis of cancer and undergoing cancer treatment and/or chemotherapy were eligible for inclusion and were recruited from 10 clinical centers across Europe. They were recruited at each participating clinical centre within 8 weeks of their initial diagnosis or disease relapse. Participant recruitment was conducted between April 2022 and October 2024. Participants in the FORTEe trial were randomly assigned to either the experimental group, which received the PEx intervention, or the control group, which received usual care. The exercise intervention lasted 8 to 10 weeks, depending on the participant's treatment schedule (31).

Assessments were conducted at baseline (T0), immediately after the intervention (T1), and at three follow-up time points (T2–T4). The primary outcome of the FORTEe trial is cancer-related fatigue (CRF), assessed using the age-specific PedsQL^TM^ 3.0 Multidimensional Fatigue Scale, completed by participants and/or their parents or guardians. Additionally, blood samples will be collected for immunological and epigenetic biomarker analyses. Following enrolment and completion of the baseline pre-test (T0), a centralized, computerized, stratified randomization allocates participants in a 1:1 ratio to the exercise or control group. Stratification was based on recruiting center, cancer diagnosis, treatment intensity, and baseline physical performance (Lansky/Karnofsky score) (47, 48). The FORTEe-exprot were developed in response to this need and were guided by a patient-centered and clinically feasible approach. An expert consultation involving researchers in clinical centers across consortium partners in Spain, France, Italy, Slovenia, Germany, United Kingdom, and Denmark was conducted to integrate scientific evidence with real-world clinical experience. The resulting FORTEe-exprot were standardized yet flexible, allowing implementation across different clinical environments, including gyms, hospital wards, and outpatient clinics. Each exercise included alternative versions specifically adapted for the frailest children and adolescents, ensuring safety, inclusivity, and continuity of training throughout the disease trajectory.

The FORTEe-exprot were disseminated through in-person training sessions for exercise professionals inside the European consortium. In addition, a paper-based booklet of the protocols provided a practical reference tool for exercise professionals.

The rationale for disseminating this methodology is to enable other research groups and clinical centers to adopt, adapt, and critically evaluate these protocols in different settings and contribute to the development of a robust evidence base for precision-based exercise interventions during intensive cancer treatment.

### Barriers to exercise participation in CAYA-c

Cancer treatment is associated with fluctuations of symptoms and moods during the course of the treatment that can greatly influence an individual's motivation to train, requiring experienced exercise professionals that can promptly adapt the level of exercise in response to individual clinical conditions (27, 49–54).

Cancer-related fatigue, pain, and acute or critical clinical conditions (e.g., nausea, diarrhea, mucositis, isolation due to resistant bacteria) constitute some of the most significant barriers to the initiation and maintenance of PEx in CAYA-c in hospital. Consequently, PEx must be individually tailored, continuously reassessed, and seamlessly integrated into clinical care, with exercise quality, intensity, and supervision adjusted in real time to ensure safety while preserving continuity whenever possible.

In pediatric oncology, concerns and potential fears related to the safety and physical demands of exercise may influence the acceptance of exercise interventions among patients, families, and healthcare professionals even after cancer treatment discontinuation (49, 50, 55). In daily clinical practice, exercise professionals frequently encounter parental and health professionals concerns about the risk of falling when exercising, or other prejudices (e.g., “exercise is not a priority for CAYA-c”; “CAYA-c are medically fragile children, not athletes”; “CAYA-c can't climb stairs”; “CAYA-c can't exercise outdoors”; “my child is tired, he/she can't exercise today”; “you can't train CAYA-c when they are on oxygen”). Together, these recurring perceptions create a multifaceted scenario that must be considered when organizing PEx programs in hospital settings.

In younger children, the main influencing factor in exercise programs participation is family involvement and support, while in adolescents, external figures such as peers, hospital staff, and the community, can play an equally crucial role. The inclusion process, in this context, requires the commitment of various actors, including physicians, nurses, psychologists, social workers, and, last but not least, the parents themselves. There is thus a need to promote a cultural change that encourages access to and participation in physical activity and sports, allowing everyone to actively participate, regardless of physical ability or age. In Figure 1 key factors limiting PEx in CAYA-c are shown.

**Figure 1:**
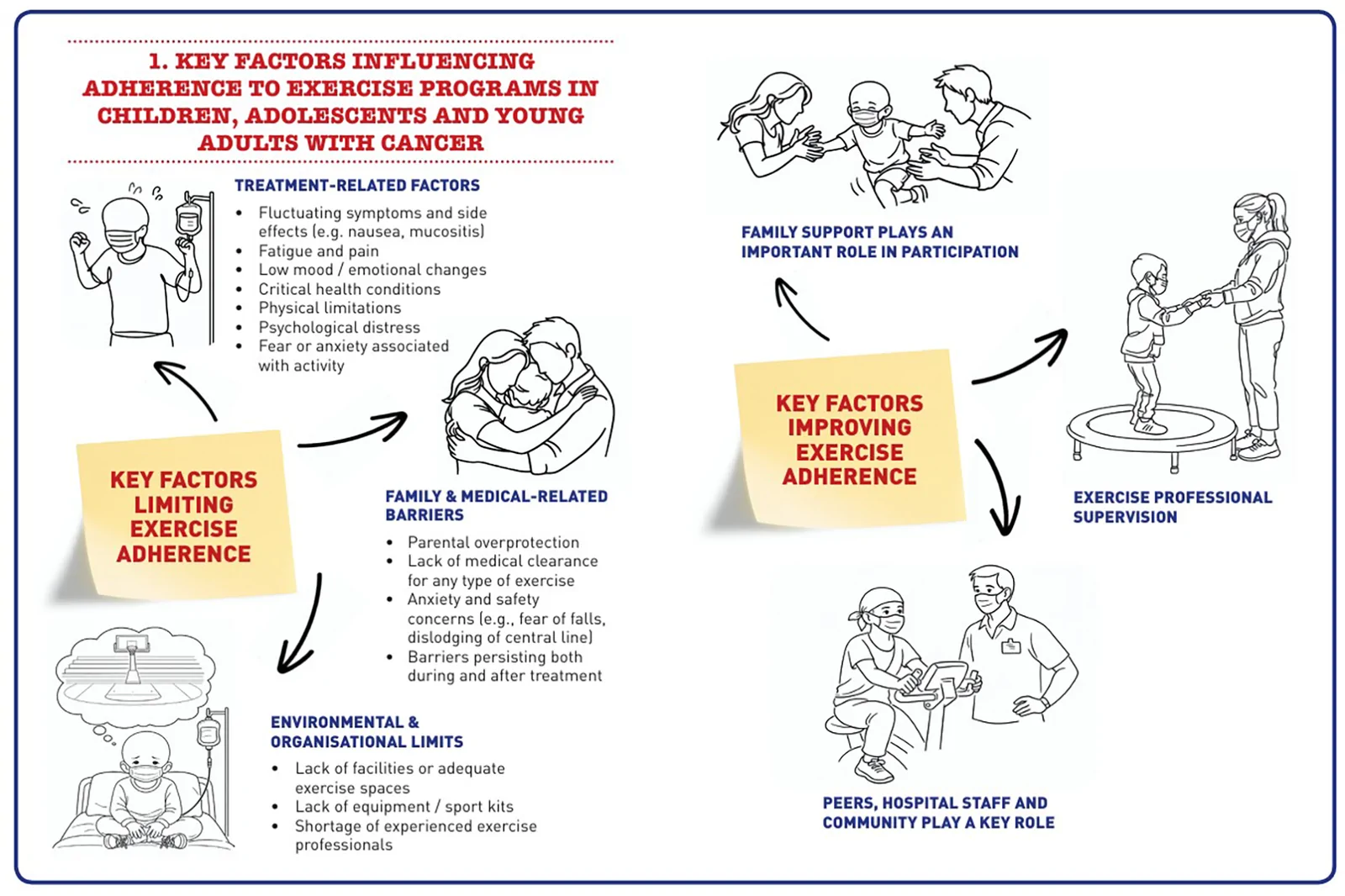
Multidimensional factors influencing adherence to exercise programs in children, adolescents, and young adults with cancer, including individual, clinical, family, and healthcare-related determinants.

In addition to these psychosocial and cultural barriers, structural limitations, such as the lack of dedicated facilities, insufficient exercise equipment, and limited availability of qualified exercise professionals with oncology-specific expertise, may represent further constraints that can substantially hinder the implementation and sustainability of hospital-based PEx programs.

### FORTEe exercise protocol: facilitating access to exercise during hospitalization

As highlighted in the “WHO global disability action plan 2014–2021,” disability is a global public health issue, with people facing barriers to healthcare and poorer outcomes worldwide. The plan calls for improved access to rehabilitation, assistive devices, community-based support, and better comparable data. Inclusive PEx can help address this by generating robust data on physiological and social outcomes an aim supported by the FORTEe project (31).

## Materials and equipment

The basic set of exercise equipment to implement the FORTEe-exprot included resistance bands, free weights, medicine balls, balance equipment, mats, and Swiss balls. In centers with more advanced infrastructure and greater experience in exercise interventions, additional equipment such as leg-extension machines and cycle ergometers was available. In these settings, vital parameters and exercise intensity were additionally monitored using a pulse oximeter, besides the Borg ratings of perceived exertion and recorded training loads and exercise intensities. This variability was acknowledged from the outset of the project, with the aim of progressively harmonizing and strengthening the available resources across centers during and beyond the FORTEe research program.

The exercise equipment was designed to meet the strict hygienic requirements of an oncological setting, where sanitation protocols must be rigorously followed. To ensure inclusivity for the most vulnerable children, the equipment should be transportable to the child's room and adaptable to different body sizes.

Motivational materials, such as stickers and paper-based tools designed for interactive activities (e.g., stitching or task completion), were also implemented to enhance engagement, reinforce positive experiences, and support adherence to the exercise sessions, particularly among younger participants (see Supplementary Material S1).

## Methods

### Ethical statement

The protocols described in this manuscript were implemented as part of a study involving human participants that received approval from the appropriate institutional ethics committee. The FORTEe study protocol and associated documents were approved by the Ethics Committee of the Rhineland-Palatinate Chamber of Physicians, the consortium's lead ethics committee, on 4^th^ August 2021 (application number 2021-15904), followed by approvals from local ethics committees and regulatory authorities at each recruitment center in accordance with national and/or local requirements.

### Development of the FORTEe exercise protocols

The FORTEe exercise protocols were developed through a structured collaborative process involving one year of consultation among the 10 clinical centers across the FORTEe consortium in Europe. Weekly online meetings facilitated discussion and consensus-building among participating stakeholders. The leader of the clinical trial work package acted as the primary author of the protocols, which were subsequently reviewed and validated by the coordinator of the European project. This iterative process resulted in the final FORTEe-exprot, ensuring a standardized yet adaptable approach suitable for different clinical settings, levels of experience among exercise professionals, and available facilities.

Recruitment was planned to start during the initial phase of cancer treatment, when CAYA-c typically receives more intensive treatment and, if possible, fully integrated into the clinical care through continuous consultation with nurses and medical staff, which worked together with exercise professionals to obtain the families commitment. Nurses play a key role within the multidisciplinary FORTEe framework by supporting symptom monitoring, safety assessment, child and family education, and communication among children, families, physicians, and exercise professionals. Their continuous involvement in pediatric oncology care contributes to the identification of clinical changes and the adaptation of exercise according to individual needs and treatment-related conditions. The multidisciplinary team and family alliance played a crucial role in the successful implementation of exercise programs in many FORTEe centers.

### FORTEe exercise protocol intensity

The FORTEe-exprot comprise endurance, strength, flexibility, and coordination/balance and gait training and are tailored to each patient's age, functional status, goals and phase of cancer treatment. Additionally, playful games are integrated into the sessions to increase motivation and enhance adherence, particularly among younger children.

Functional status is assessed using the Karnofsky Performance Status for adults and the Lansky Play-Performance Scale for children (47, 48), while cancer treatment intensity was classified according to the Intensity of Treatment Rating Scale–3 (ITR-3) (56).

Three to 5 exercise sessions per week are planned at moderate-to-vigorous intensity, with frequency and intensity adapted to the clinical condition of the CAYA-c. Digital tools and telehealth solutions support the intervention, allowing for both in-person and remote training.

Exercise training is delivered across inpatient and outpatient clinical settings as well as in patients' homes via online platform during outpatient stays. During inpatient admissions, sessions are directly supervised by qualified exercise professionals, whereas during outpatient periods patients are trained following individualized exercise recommendations provided by FORTEe exercise professionals. Remote outpatient training can also be offered via GDPR compliant telehealth solutions under professional supervision. Session duration is intended to be last 45 to 60 min and is individualized according to the patient´s functional capacity, age, fitness level, and overall health status.

The participant-to-exercise professional ratio ranges from 9:2 to 1:1, depending on clinical needs. For each session, total exercise duration and time spent in each exercise category is documented; session cancellations or early terminations and their reasons are also recorded. The characteristics of the training protocols according to ITR-3 classification are summarized in Figure 2.

**Figure 2:**
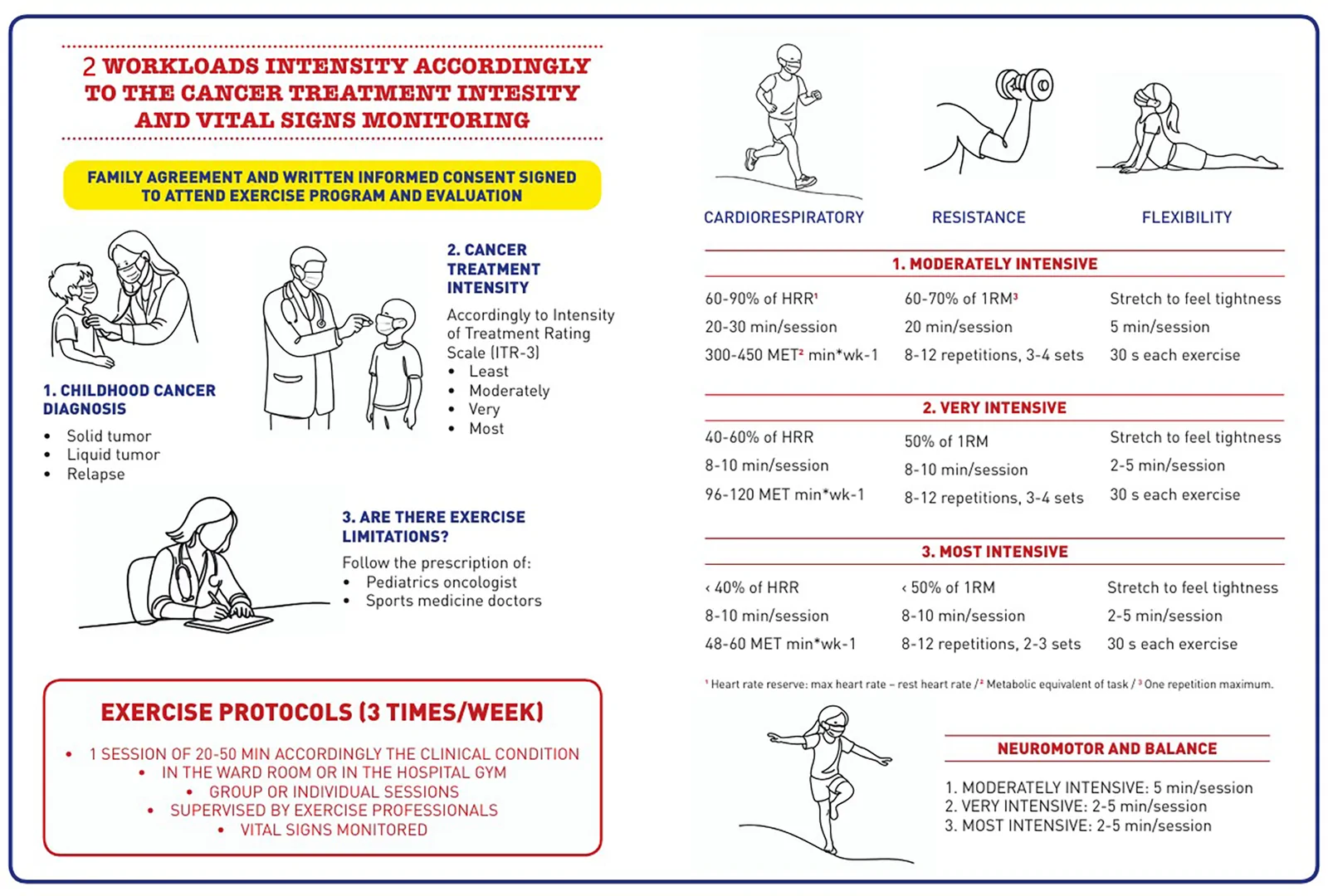
Framework for adapting exercise workload according to cancer treatment intensity, clinical status, and vital signs monitoring-.

In Table 1 the major components of the FORTEe-exprot, with aims, physiological targets and treatment modalities are shown.

**Table 1:** Components of the FORTEe exercise protocol.

Training Component	Aim	Physiological Targets	Examples of Exercises/Modalities in FORTEe exercise protocol
Endurance	Improve aerobic capacity and exercise tolerance	Pulmonary ventilation, cardiac output, peripheral oxygen extraction	Walking, trampoline walking, cycling
Strength	Enhance muscular strength and functional capacity	Neuromuscular activation, muscle fiber recruitment, musculoskeletal adaptations	Upper and lower body exercises targeting legs/hips, back, chest, shoulders, arms, and core using body-size–adaptable machines, dumbbells, elastic (resistance) bands, and body-weight exercises
Gait exercise	Improve postural control and stability	Integration of visual, vestibular, and proprioceptive inputs	Exercises on unstable surfaces (proprioceptive cushions, trampolines) in single and double leg positions with eyes open or closed
Coordination/balance	Enhance neuromuscular coordination and motor control	Motor planning and execution	Playful activities using balls of different sizes and weights
Flexibility	Improve joint range of motion and muscle extensibility	Muscle elasticity and connective tissue mobility	Stretching exercises targeting large muscle groups, primarily performed at the end of the session

Potential clinical conditions in which exercise should be avoided are carefully considered to ensure safety. A comprehensive list of such conditions, along with adapted training sessions for children with serious medical complications, is provided in Supplementary Material S2, and they are part of a specific chapter of the downloadable protocols. The criteria are based on the FORTEe study design (31) and the Italian partner MBBM proposed site-specific adaptations according to local clinical practice and logistical considerations; similarly, other centers may implement comparable adaptations based on their own clinical protocols and organizational requirements. Exercise intensity and progression were individualized according to the participant's clinical condition and functional status. Before each session, exercise professionals assessed treatment-related symptoms, vital signs, fatigue, pain, and overall well-being and motivation to determine readiness for exercise. Intensity and workload were adjusted based on perceived exertion, functional capacity, and participant tolerance. In CAYA-c with known or suspected treatment-related clinical conditions (e.g., anemia, osteonecrosis, or bone marrow aplasia), the exercise program was adapted accordingly, including specific precautions such as avoidance of heavy cardiopulmonary workloads, isolation measures when required. Exercise progression was implemented only when participants tolerated the prescribed activities without adverse symptoms or excessive fatigue. Exercise intensity was reduced or activities were modified in the presence of relevant symptoms, abnormal vital signs, acute illness, or other contraindications, according to the study safety protocol. To facilitate safe and consistent exercise execution, each exercise is described in detail within the protocol, including (as an example see Supplementary Material S3):

1. Starting position, specifying the correct initial posture;
2. Action, describing the proper execution of the exercise;
3. Time, detailing duration and rest;
4. Breathing, indicating the correct timing for exhalation and inhalation;
5. Stabilization, providing instructions for maintaining postural control and highlighting actions or postures that should be avoided to reduce risk of injury or overexertion.

In line with the guiding philosophy of the FORTEe research project “Never avoid exercise, just adapt it” the FORTEe-exprot emphasizes both the safety and empowerment of CAYA-c, ensuring that even the most vulnerable can participate in structured, motivating, and therapeutically beneficial exercise sessions.

### Risk management

Figure 3 summarizes the main risks highlighted through a structured expert consultation conducted one year prior to the start of the clinical trial with the consortium's leading specialists in pediatric oncology exercise, along with the preventive measures implemented to mitigate these risks.

1. Risk of injury or training overload
  (a) Mitigation strategies included careful control of training load, close supervision by specially qualified and trained study staff, and use of exercise equipment adaptable to the physiological size of children and adolescents.
  (b) Reasons for adaptation and stopping criteria were clearly defined for both testing and training, including medical clearance prior to participation.
2. Risk of insufficiently experienced staff
  (a) To minimize this risk, monitoring procedures included site visits to each clinical center (remote or in-person).
  (b) Study staff received explicit training on inclusion/exclusion criteria and protocol requirements.
3. Risk of low data quality
  (a) Experienced exercise professionals and scientists developed standardized testing and intervention procedures.
  (b) Standardized methods and equipment were used across all centers.
  (c) Study procedures, equipment and facilities were closely monitored, particularly with regard to exercise interventions.
  (d) Additional staff training was provided as needed to maintain high data quality.
4. Risk of low recruitment
  (a) Recruitment was promoted using leaflets and technology, targeting the interests of children, adolescents, and young adults.
  (b) If recruitment fell below expectations, additional promotion strategies and extending recruitment to other FORTEe centers were planned.
  (c) Sufficient lead time (“run-up”) was allowed to support enrollment.
5. Risk of low compliance by pediatricians
  (a) Monitoring, including site visits (remote or in-person), was performed to ensure protocol adherence.
  (b) Explicit training for study staff on trial procedures and responsibilities was conducted.
  (c) In cases of non-compliance, additional monitoring or shifting recruitment to other clinical sites was considered.

**Figure 3:**
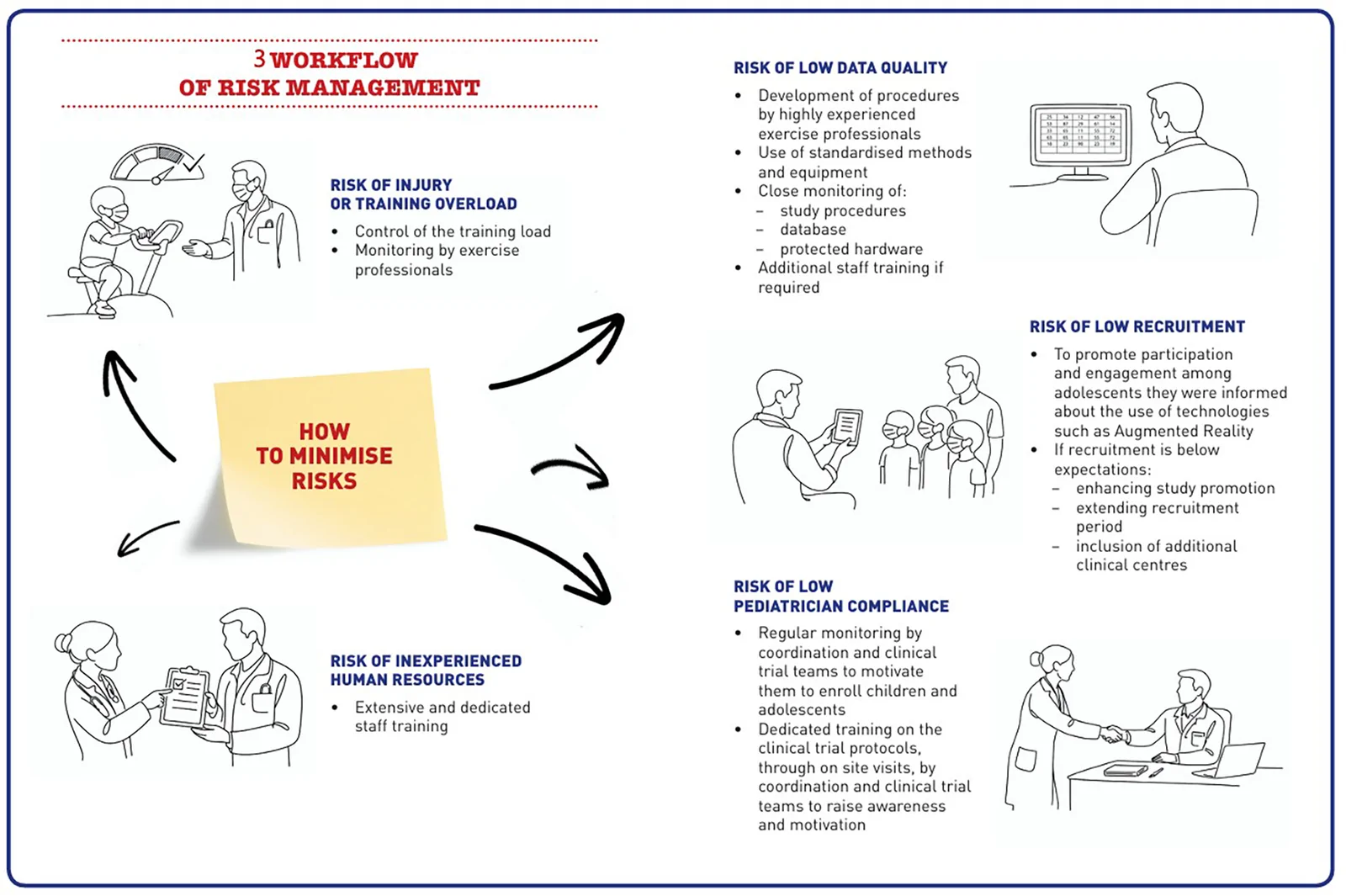
Workflow for clinical risk assessment and exercise adaptation during the FORTEe intervention.

## Results

No clinical outcomes from the FORTEe randomized controlled trial are reported in this manuscript, as the focus of this work is the methodological development, application, and dissemination of the FORTEe-exprot.

A final version of the FORTEe-exprot was completed by March 2022, prior to the start of clinical trial recruitment. During their development, potential risks, including injury, staff experience, data quality, recruitment, and compliance, were systematically considered and addressed through expert consultation and the adoption of preventive measures. The FORTEe exercise protocols were designed to be inclusive, accommodating children with different clinical needs and functional capacities while ensuring applicability across clinical centers with varying levels of expertise among exercise professionals and diverse available facilities.

### Dissemination

The FORTEe-exprot and associated implementation procedures were applied consistently to integrate PEx into diverse clinical settings. In addition, the methodology was disseminated within the consortium to ensure harmonized application across sites and to provide a practical reference for exercise and healthcare professionals. The latter, including doctors, psychologists and nurses, played a pivotal role in integrating the project into clinical routines. The FORTEe researchers began sharing the internal exercise protocols as a methodological resource. A printed handbook of the protocols was produced, and the scientific community was engaged through scientific conferences (2 symposia, 1 plenary session, 6 scientific meetings presentations, and participation in collaborative research networks). A total of around 500 handbooks of the FORTEe-exprot were distributed at two major international pediatric oncology congresses: the SIOP Europe Annual Meeting held in Budapest in 2025 and the International Society of Paediatric Oncology (SIOP) Congress held in Amsterdam in 2025. Through these events, the materials reached healthcare professionals from 50 countries across five continents. Pediatricians and specialists in physical medicine and rehabilitation played a central role in the dissemination process by introducing and reporting on the FORTEe exercise protocols within their respective clinical centers. In addition, family associations and childhood cancer survivor associations also received copies of the protocols and expressed strong enthusiasm for the FORTE program, highlighting its perceived relevance and practical value for patients and survivors.

Dissemination was also fostered by other outreach strategies including social media campaigns on platforms such as Instagram, Facebook and LinkedIn, press releases for key milestones and participation in podcasts, interviews and local media features. These strategies were all aimed at amplifying the impact of FORTEe exercise protocols. Short videos and infographics were used to explain the benefits and safety of exercise in paediatric oncology to non-specialist audiences and finally policy makers and institutions were engaged through policy briefs summarizing the FORTEe exercise protocols, targeted meetings with hospital staff meetings for the integration of structured exercise programs into standard paediatric oncology care.

By combining these channels of dissemination, the FORTEe exercise protocol maximized its impact, fostered engagement across multiple levels, and created the conditions for sustainable implementation and replication in other centers (Figure 4).

**Figure 4:**
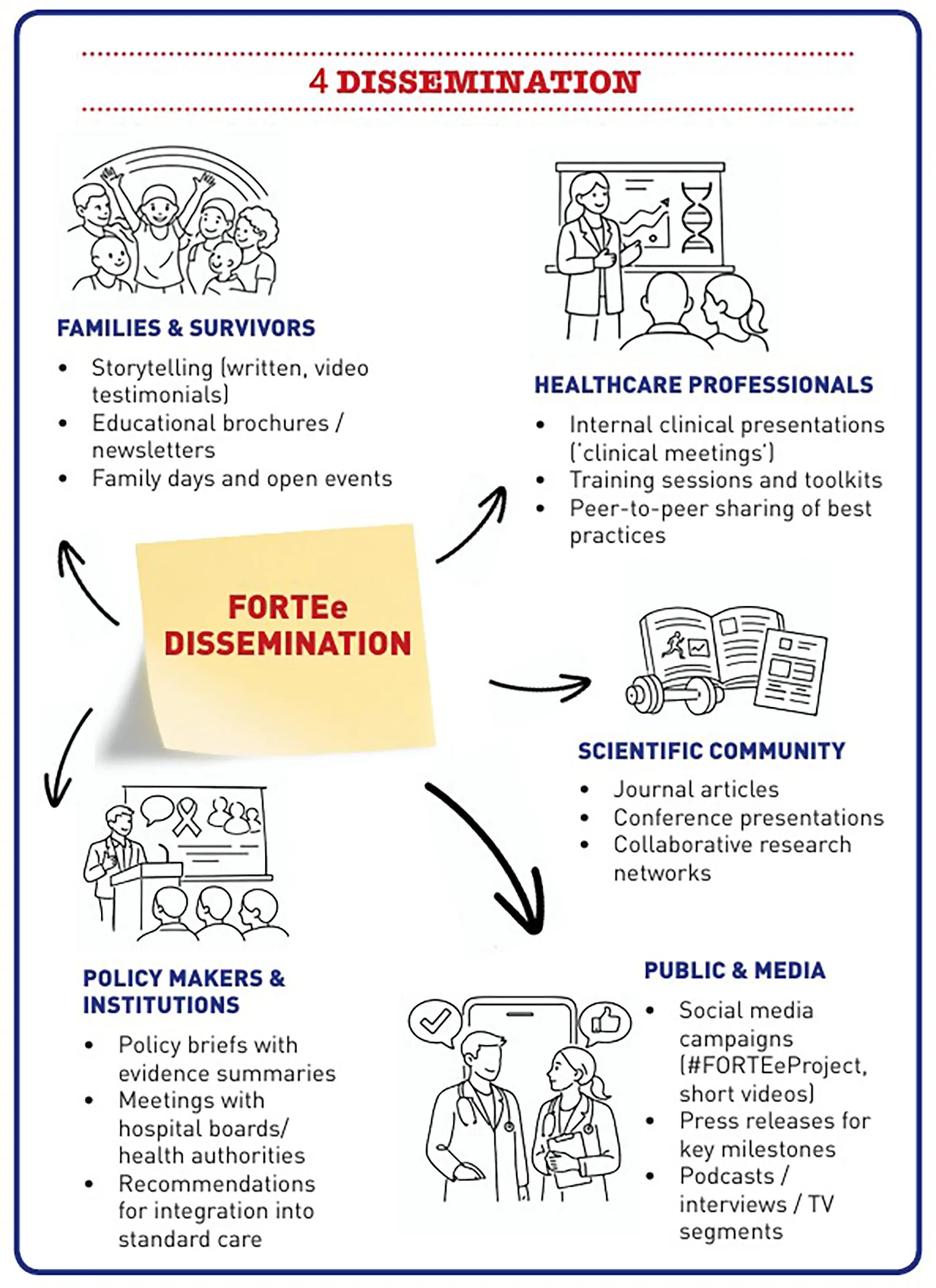
FORTEe dissemination strategy to support the implementation and adoption of exercise programs in pediatric oncology settings.

## Discussion

Effective implementation of PEx requires appropriately trained exercise professionals, risk-stratified prescription, systematic documentation, and close interdisciplinary collaboration to ensure safe and individualized interventions aligned with each child's clinical status. This multi-stakeholder collaborative approach constituted a key foundation of the FORTEe-exprot, highlighting their potential role in supporting pediatric oncology care and their possible contribution to the development of future evidence-based recommendations.

Unlike in adult oncology, where comprehensive exercise guidelines exist (57, 58), pediatric oncology currently lacks evidence-based recommendations. Adult-focused documents explicitly caution against direct translation, given the limited pediatric data. The FORTEe-exprot were developed as a standardized framework for individualized exercise interventions in CAYA-c. Preliminary implementation experience suggests that the protocol is feasible and safe under trained supervision. Comprehensive analyses of participant outcomes will be presented in a subsequent scientific publication from the FORTEe project.

The implementation of innovative action plans that promote the right of CAYA-c to access PEx has the potential to foster cross-institutional collaboration. In this context, the FORTEe-exprot may serve as a valuable resource and a possible cornerstone for the development of future clinical guidelines, supported by leading international experts, key stakeholders, and scientific clinical and exercise associations.

Consistent with the framework articulated by Schmitz et al. (58) and aligned with ACSM and ESSA position statements (46), for individuals with cancer to achieve clinically meaningful benefits from exercise, programs must be based on an appropriate exercise prescription, including clearly defined dose, modality, frequency, intensity, and duration. Moreover, exercise interventions in oncology should be delivered and supervised by qualified exercise professionals with competencies in exercise science, oncology, and behavioral science, ensuring safe and effective implementation through appropriate risk stratification and clinical decision-making (59, 60). In pediatric oncology, these requirements are further amplified, as compared with adult populations, due to developmental considerations and heightened treatment-related vulnerabilities, thereby necessitating specialized human resources and enhanced risk management strategies tailored to CAYA-c. Consultation and close interdisciplinary collaboration between clinicians and qualified exercise professionals are essential to ensure accurate exercise prescription and the safe, effective, and purpose-driven implementation of physical activity interventions.

The interest expressed by pediatric cancer survivors, families, and healthcare professionals in the dissemination of the FORTEe booklet and protocols highlights the growing recognition of the need for standardized pediatric exercise approaches. Further research and implementation efforts are needed to support the integration of evidence-based PEx programs within pediatric oncology care.

## Limitations

This study has several limitations that should be considered when interpreting its findings. First, the present manuscript describes the rationale, design, and implementation of the FORTEe-exerpro; therefore, final clinical outcomes and intervention effects are not reported here and will be presented in a subsequent publication following completion of the trial analyses.

Second, although standardized procedures and training were implemented across participating centers, some variability in protocol delivery may occur due to differences in clinical settings, available facilities, equipment, and local organizational aspects. The successful implementation of the exercise intervention also relies on the availability of trained exercise professionals with expertise in pediatric oncology populations, which may limit its applicability in settings with limited resources or insufficient specialized personnel.

Furthermore, despite predefined criteria for adapting exercise intensity and progression according to treatment status, functional capacity, symptoms, and safety considerations, individual clinical characteristics may influence the implementation of the intervention. Finally, as the FORTEe trial is conducted within specialized clinical centers, the generalizability of the findings to other healthcare contexts, particularly low-resource settings, may be limited.

## Data Availability

Publicly available datasets were analyzed in this study. This data can be found here: https://fortee-project.eu/wp-content/uploads/2026/05/EXERCISE-definitivo-con-cover-ottimizzato.pdf.

## Supplementary material

The Supplementary Material for this article can be found online at: https://www.frontiersin.org/articles/10.3389/fped.2026.1907775/full#supplementary-material

Supplementary Data Sheet 1 Motivational materials to improve exercise adherence

Supplementary Data Sheet 2 Examples of exercise prescriptions for specific clinical conditions

Supplementary Data Sheet 3 Examples of exercises and their adaptations
