# Supplementary material for: Precision-based exercise protocols for children with cancer: a methodological approach from the European FORTEe research project
Source: Front Pediatr. 2026 Sep 17;14:1907775. doi: 10.3389/fped.2026.1907775 (PMC13627370; doi:10.3389/fped.2026.1907775)

FORTEe challenges

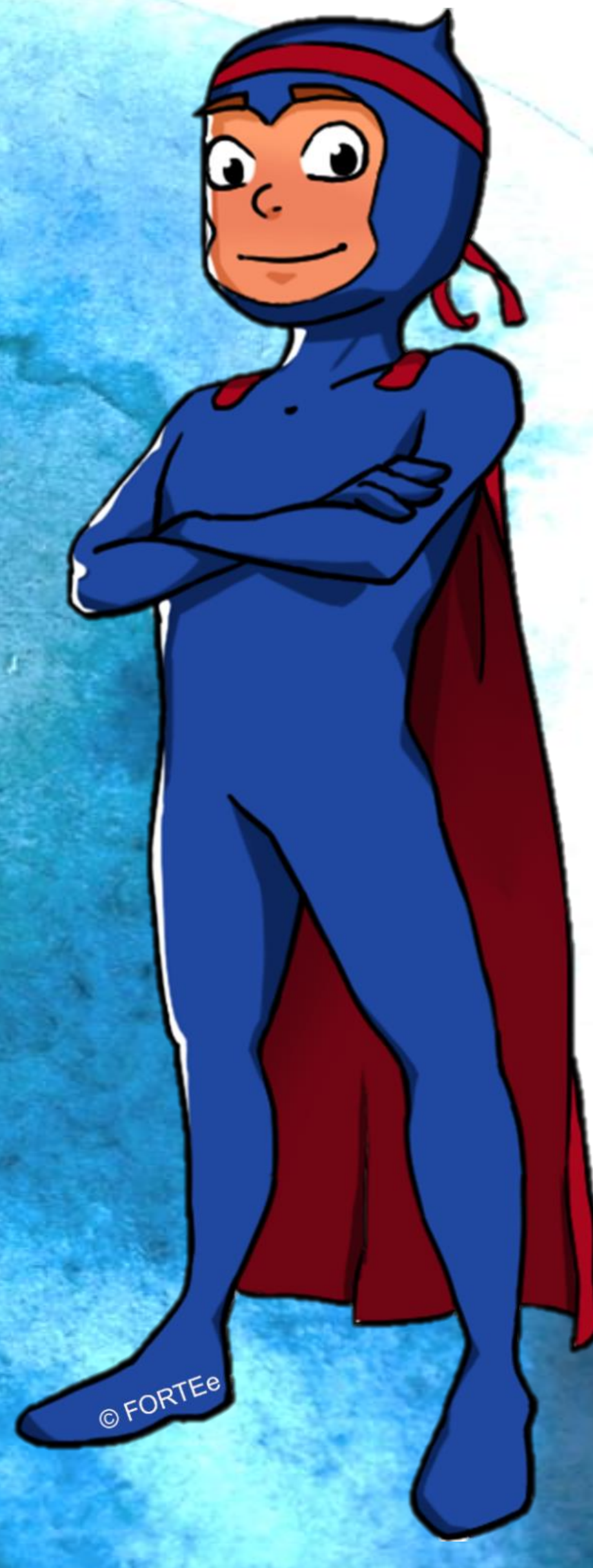

This project received funding from the European Union Research
and Innovation Programme Horizon 2020 under Grant Agreement No. 945153.

FORTEe challenges

Are you an athlete looking for a new challenge or just want to discover the FORTEe challenges?
This is the place for you!

I am **FORTEe** and I will guide you through the exercise challenges!

During your intervention and beyond, you will have the opportunity to collect stickers or stamps in this little booklet by completing small challenges.

Challenges can be either related to the general collection of stickers / stamps in your training plan, e.g. "Collect one sticker per category (endurance, strength - upper or lower body, agility, coordination / balance, gait)", or they can be related to specific exercise categories that you train with your exercise therapist, e.g. certain exercises to improve strength.

Your exercise therapist will provide you with stickers or stamps.
If you would like to try a particular challenge, just talk to your exercise therapist and they will be happy to help you and include the best exercises into your training.

Have fun and good luck with the challenges!

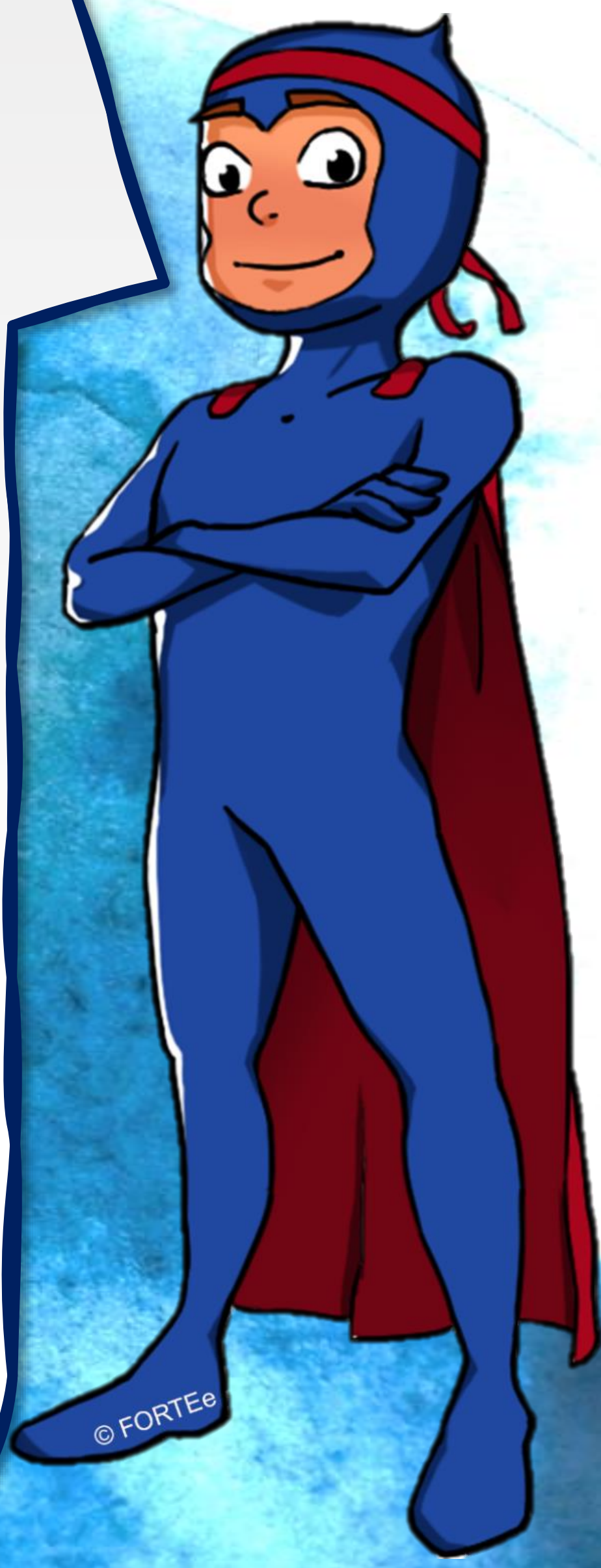

This project received funding from the European Union Research and Innovation Programme Horizon 2020 under Grant Agreement No. 945153.

FORTEe challenges

The all-rounder
Collect one sticker per category (endurance, strength - upper or lower body, flexibility, coordination / balance, gait).

All good things come in threes!
Do three sessions of cardio and three sessions of strength over two weeks.

The Athlete
Complete 5 exercise sessions in each of the 6 categories.

The machine
Collect three stickers each in the categories endurance and strength (upper and lower body) and two stickers each in the categories coordination / balance, flexibility and gait.

Keep going!
Collect at least two endurance training stickers each week.

Pump it up!
Collect at least two strength stickers each week.

Coordination
Collect at least one coordination sticker each week.

The gymnast
Collect at least one flexibility sticker each week.

I like to move it, move it…
Collect at least one gait sticker each week.

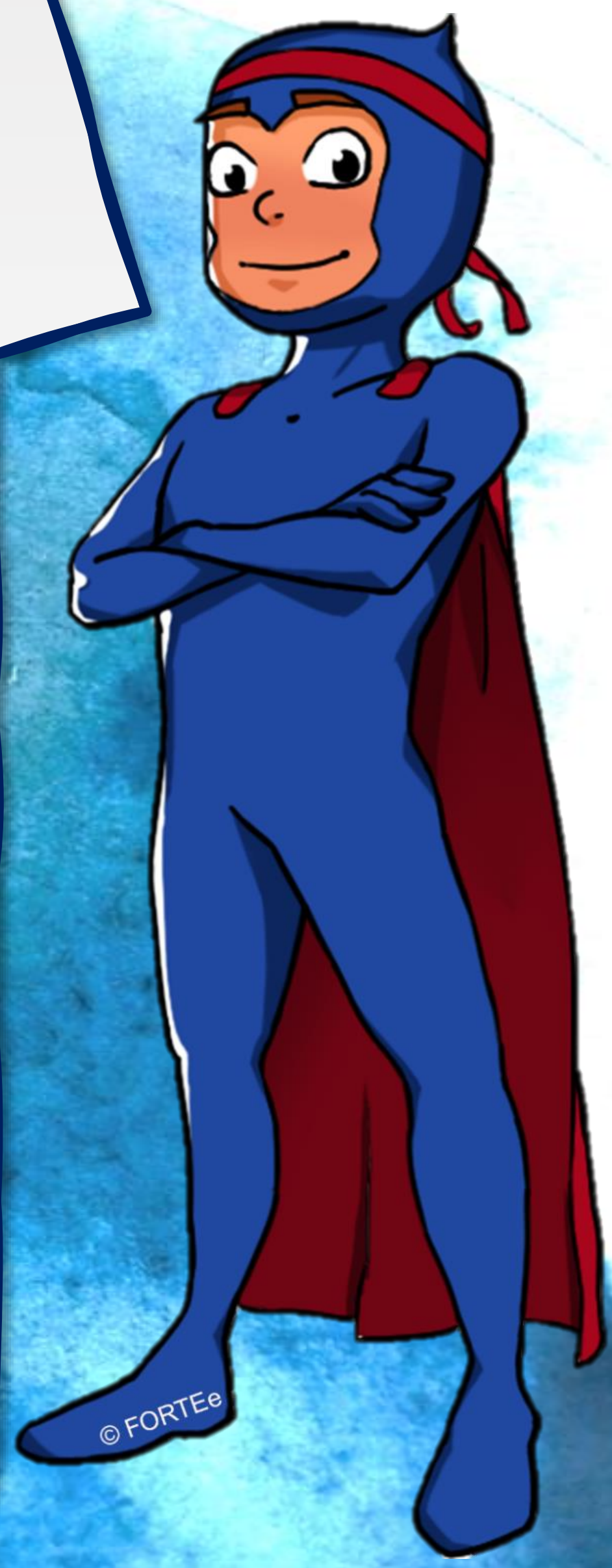

This proj
and Innovation Programme Horizon 2020 under Grant Agreement No. 945153.

FORTEe challenges

ENDURANCE

The cycling professional
Can you ride for 20 minutes on a bike ergometer/hand crank ergometer? Get your next endurance sticker here.

The half marathon
Ride a bike or rowing ergometer for a total of 21km.

The marathon
Ride a bike or rowing ergometer for a total of 42km.

Up high
Climb a total of 300 steps. You can also complete this challenge by climbing stairs in the hospital with your exercise therapist.

The walker
Make sure you go for a walk at least once a week.

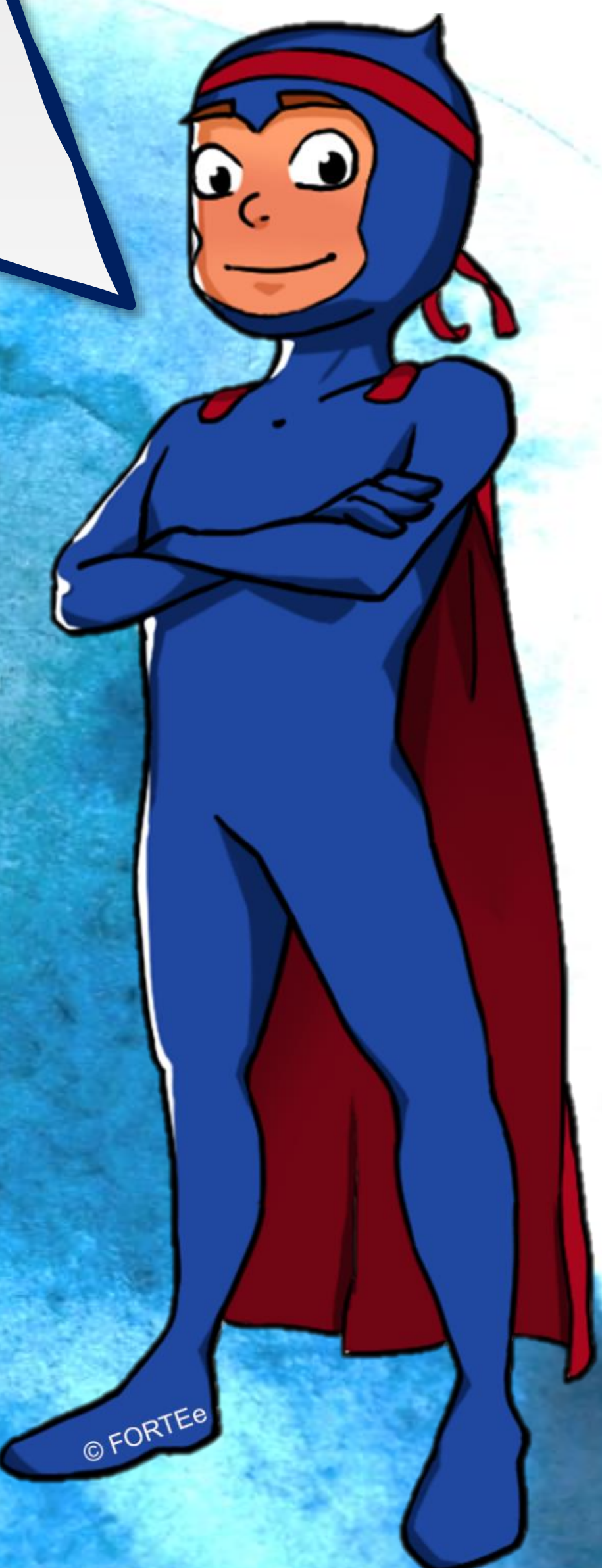

This project received funding from the European Union Research and Innovation Programme Horizon 2020 under Grant Agreement No. 945153.

FORTEe challenges

STRENGTH

The muscle pack
For three weeks in a row, collect at least two strength stickers per week.

Superhero!
Train your upper body and complete 3 exercise sessions in which you train your arms, abdomen, and back.

The strong Hulk
Train your lower body and complete 3 exercise sessions in which you train your legs.

The bodybuilder
Complete a strength workout consisting of at least 6 different strength exercises. If your strength training consists of upper and lower body exercises, you will receive a strength sticker for each.

Who has the biggest biceps?
Perform a total of 120 biceps curls over different workouts

The plank
Plank for 20 minutes over different workouts and train your whole body in a single exercise.

Leg day
Collect strength stickers and complete a total of 200 squats over different workouts.

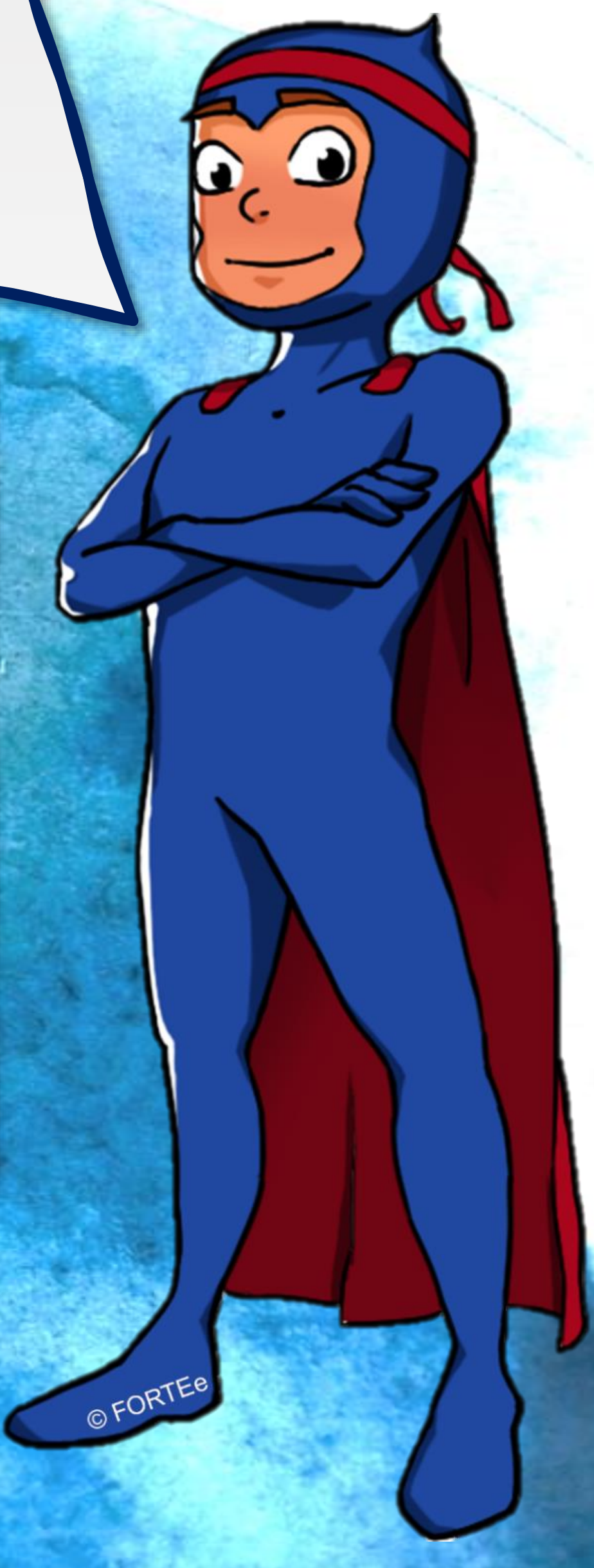

This project received funding from the European Union Research and Innovation Programme Horizon 2020 under Grant Agreement No. 945153.

FORTEe challenges

COORDINATION

„Three in a row“
Collect at least two coordination stickers for 3 weeks in a row.

☐

Juggling
Practice juggling with 2-3 balls/beanbags in at least three exercise sessions. Can you juggle three balls/beanbags after three sessions?

☐

The basketball pro
Score a total of 100 goals by shooting at a target (minimum distance: 10 steps).

☐

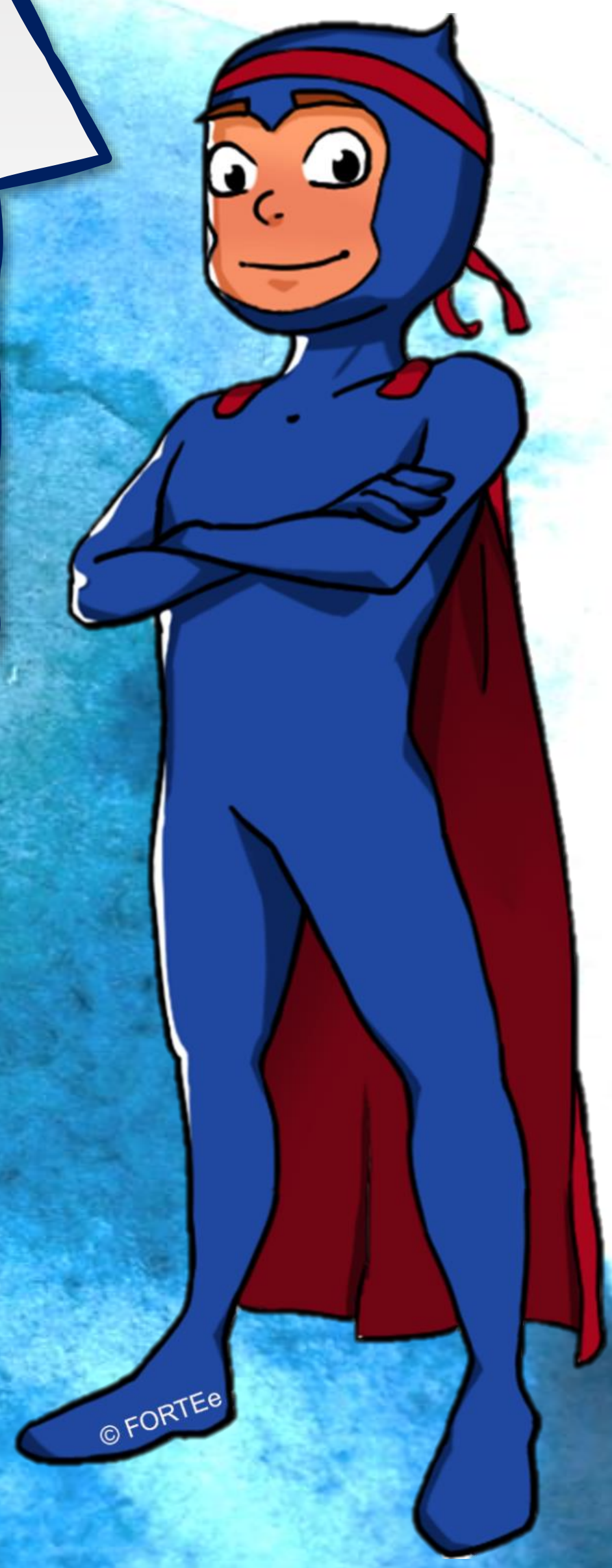

This project received funding from the European Union Research and Innovation Programme Horizon 2020 under Grant Agreement No. 945153.

FORTEe challenges

BALANCE

Tightrope walker
Balance on a wobbly surface. You and your exercise therapist are throwing a ball to each other. Can you make 15 passes without dropping the ball or touching the ground with your feet? Can you do this five times during the intervention?

The Flamingo
Try to stand on one leg for a total of 10 minutes over the course of a week. You have completed the challenge if you can do the exercise with both your right and left leg.

The balancer
Train your balance at least once a week for a total of 8 weeks (either in a gym session with your exercise therapist or at home, standing on one leg while brushing your teeth).

The floor is lava
The floor is lava and must not be touched. Try to move around the gym or garden for at least 15 minutes without touching the floor.

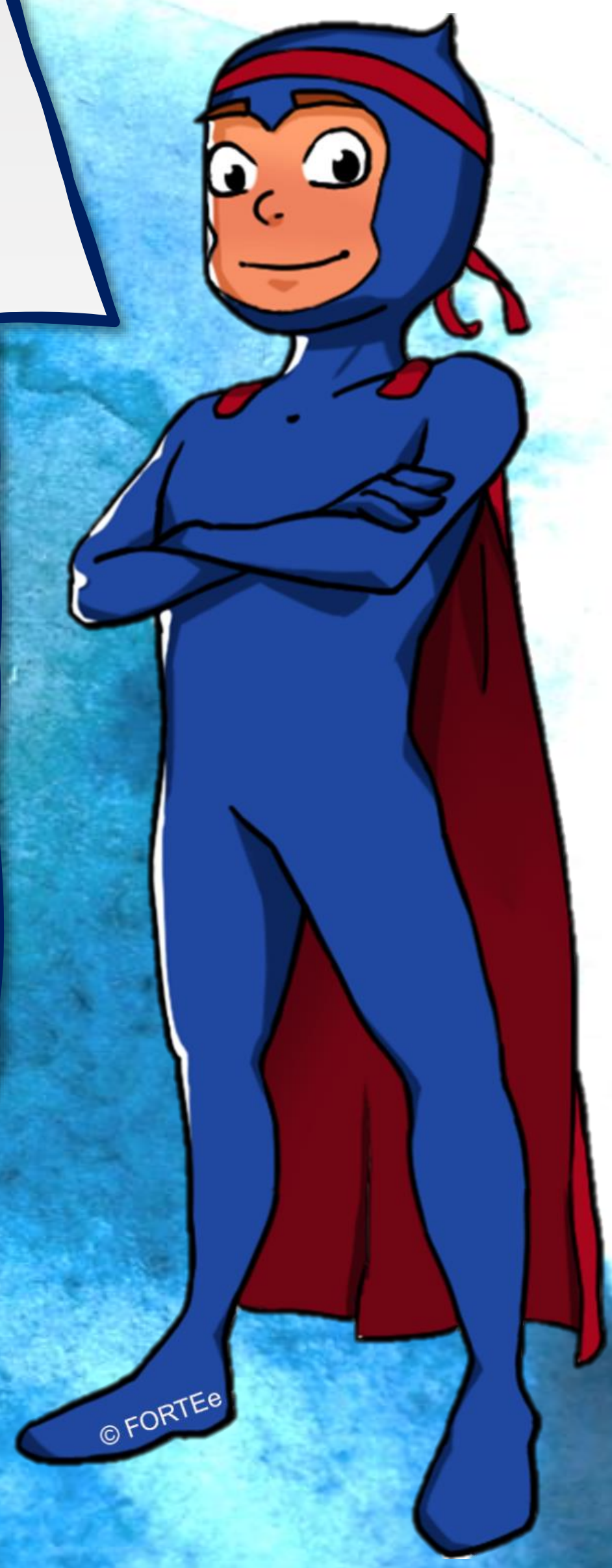

This project received funding from the European Union Research and Innovation Programme Horizon 2020 under Grant Agreement No. 945153.

FORTEe challenges

FLEXIBILITY

Bendy
Improve your mobility during the intervention phase. After 8-10 weeks of training, can you touch your knees with your nose without bending your legs? OR Can your fingers touch your toes without bending your legs? ☐

Mr Mobility and Miss Elasticity
Train your flexibility three times in two consecutive weeks. ☐

The elastic human
Complete 10 training sessions with relaxing stretching exercises. ☐

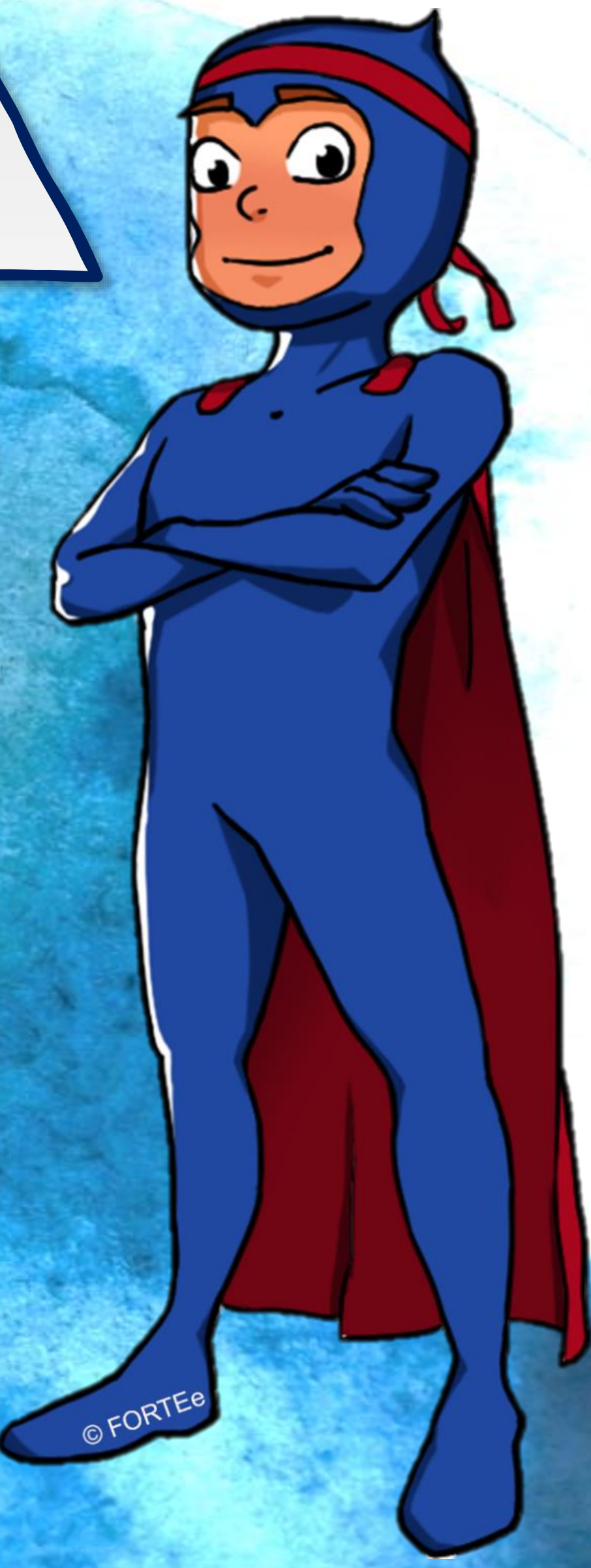

This project received funding from the European Union Research and Innovation Programme Horizon 2020 under Grant Agreement No. 945153.

FORTEe challenges

GAIT

The Elephant and Mouse
Take giant steps like an elephant, tiny steps like a mouse, sneak like a lion or stomp like a rhino. Can you imitate 10 animals? If your exercise therapist can recognise at least 8 animals, you will receive this sticker/stamp.

The champion
Get to 6,000 steps three times a week. Use a smartwatch, your smartphone or ask your exercise therapist for an activity tracker to count your steps.

The runway
Walk a total of 75 km during the period of the exercise intervention. Use a smartwatch, your smartphone or an activity tracker to measure the distance covered.

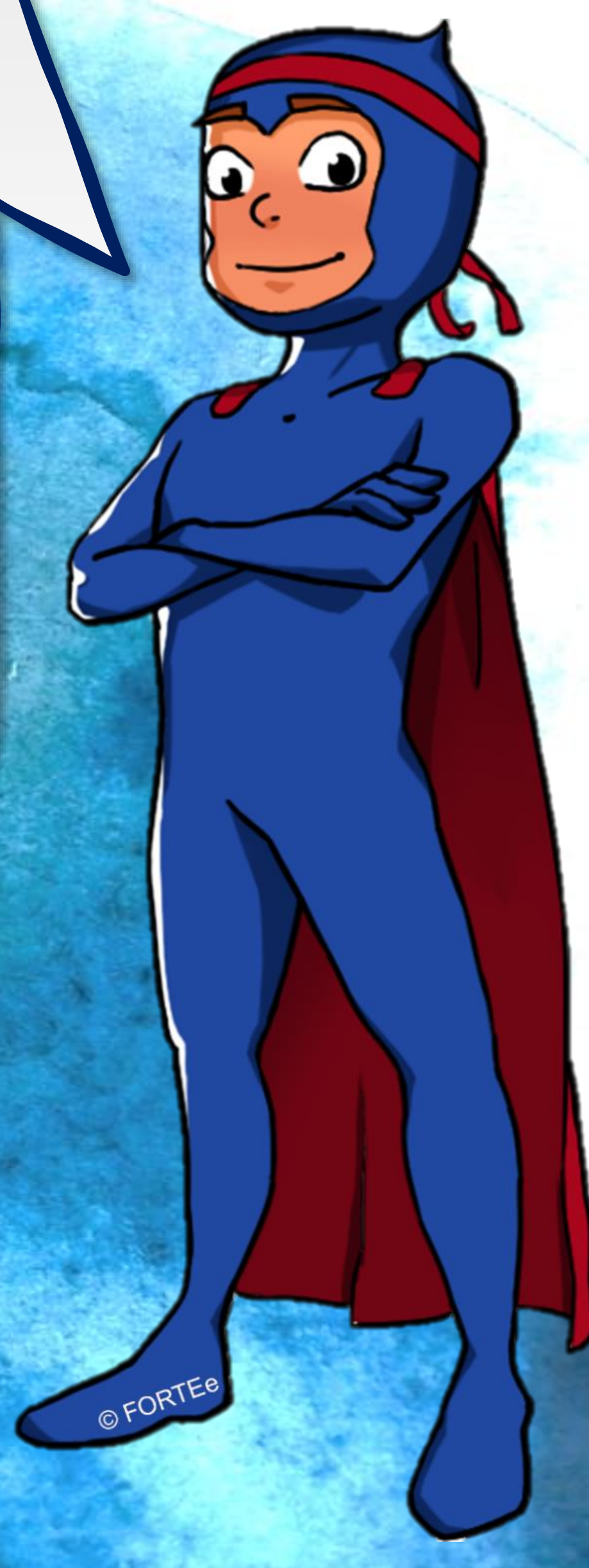

This project received funding from the European Union Research and Innovation Programme Horizon 2020 under Grant Agreement No. 945153.

The great FORTEe Scrapbook

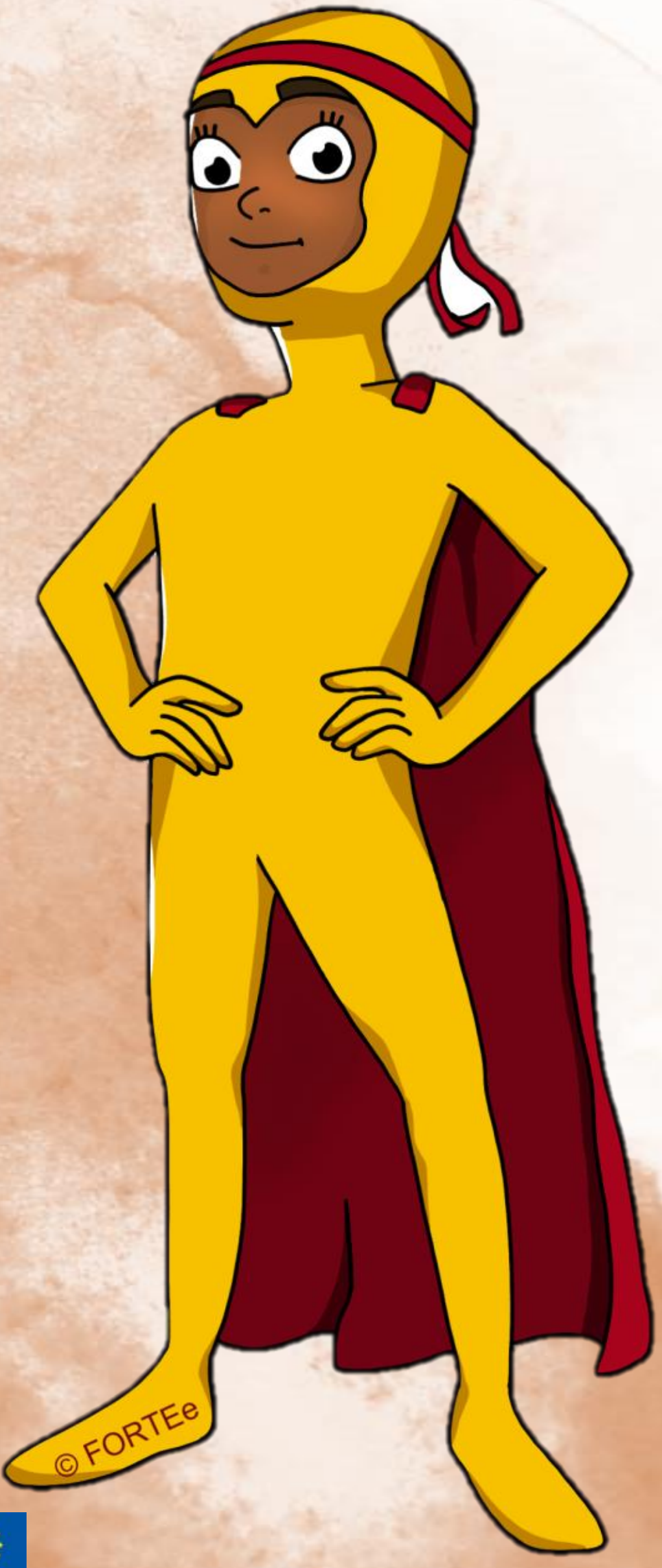

This project received funding from the European Union Research and Innovation Programme Horizon 2020 under Grant Agreement No. 945153.

The great FORTEe Scrapbook

Welcome to the FORTEe exercise group!

I'm **Coordi** and I'll be with you during the 8-10 week intervention, along with the other superheroes.

In this booklet you will be able to document your exercise sessions with your exercise therapists.

Your **FORTEe training** will consist of different exercises in the categories of **endurance**, **strength**, **flexibility**, **coordination** and **balance**, as well as **gait**.

At the end of each session, your therapist will award you with stickers or stamps for each category you have been working on.

There are up to 5 training sessions per week, so if you do endurance training 5 times a week, for example, you will receive 5 endurance stickers for that week.
The fields with a darker background show you how many times a week you should ideally train in this category.

Have fun training and collecting stickers!
See you soon!

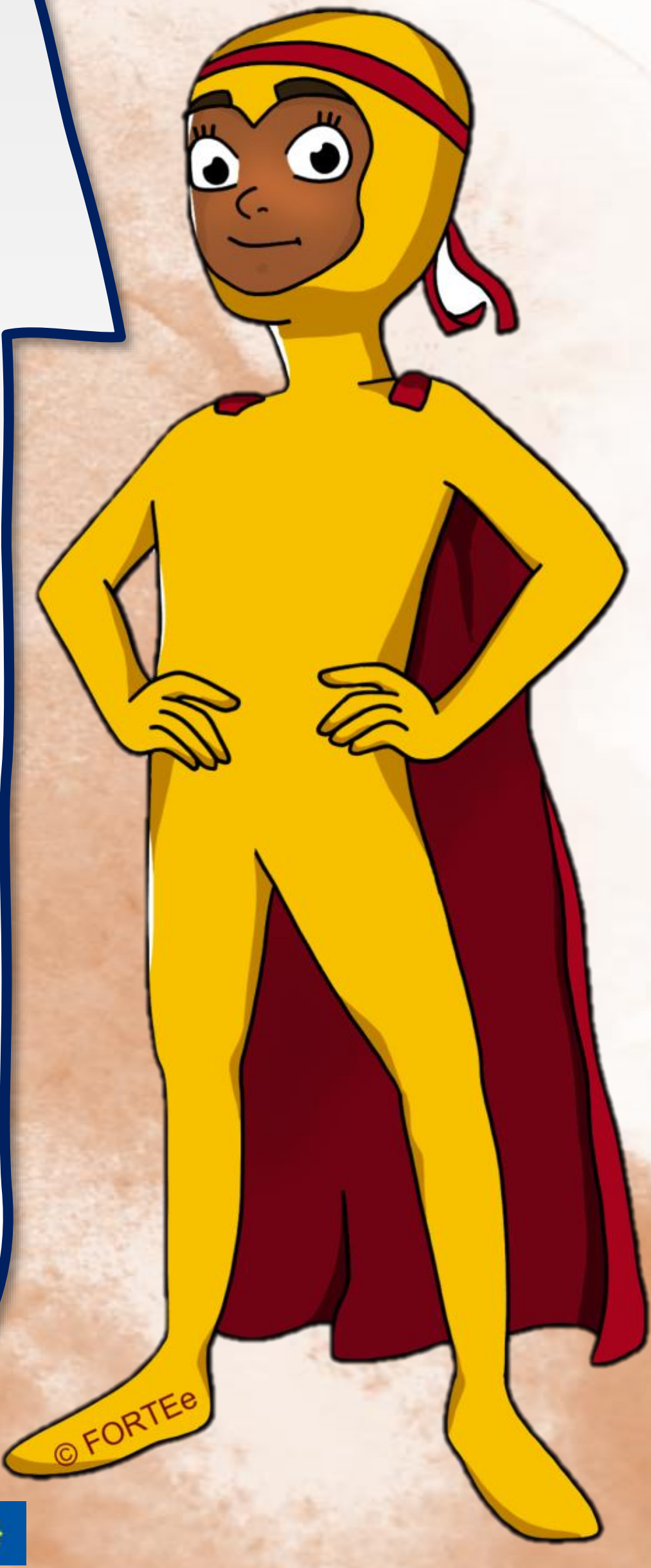

This project received funding from the European Union Research and Innovation Programme Horizon 2020 under Grant Agreement No. 945153.

Endurance

Week 1					
Week 2					
Week 3					
Week 4					
Week 5					
Week 6					
Week 7					
Week 8					
Week 9					
Week 10					

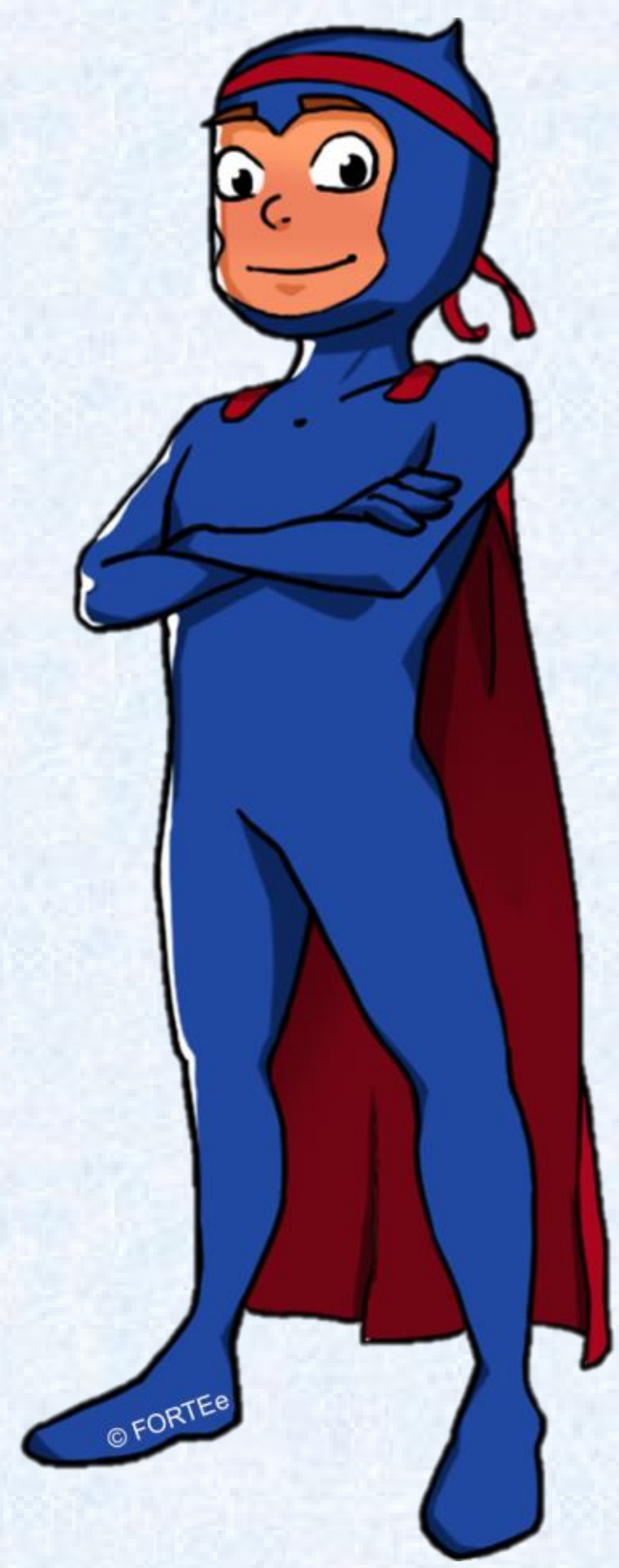

Strength – Upper body

Week 1					
Week 2					
Week 3					
Week 4					
Week 5					
Week 6					
Week 7					
Week 8					
Week 9					
Week 10					

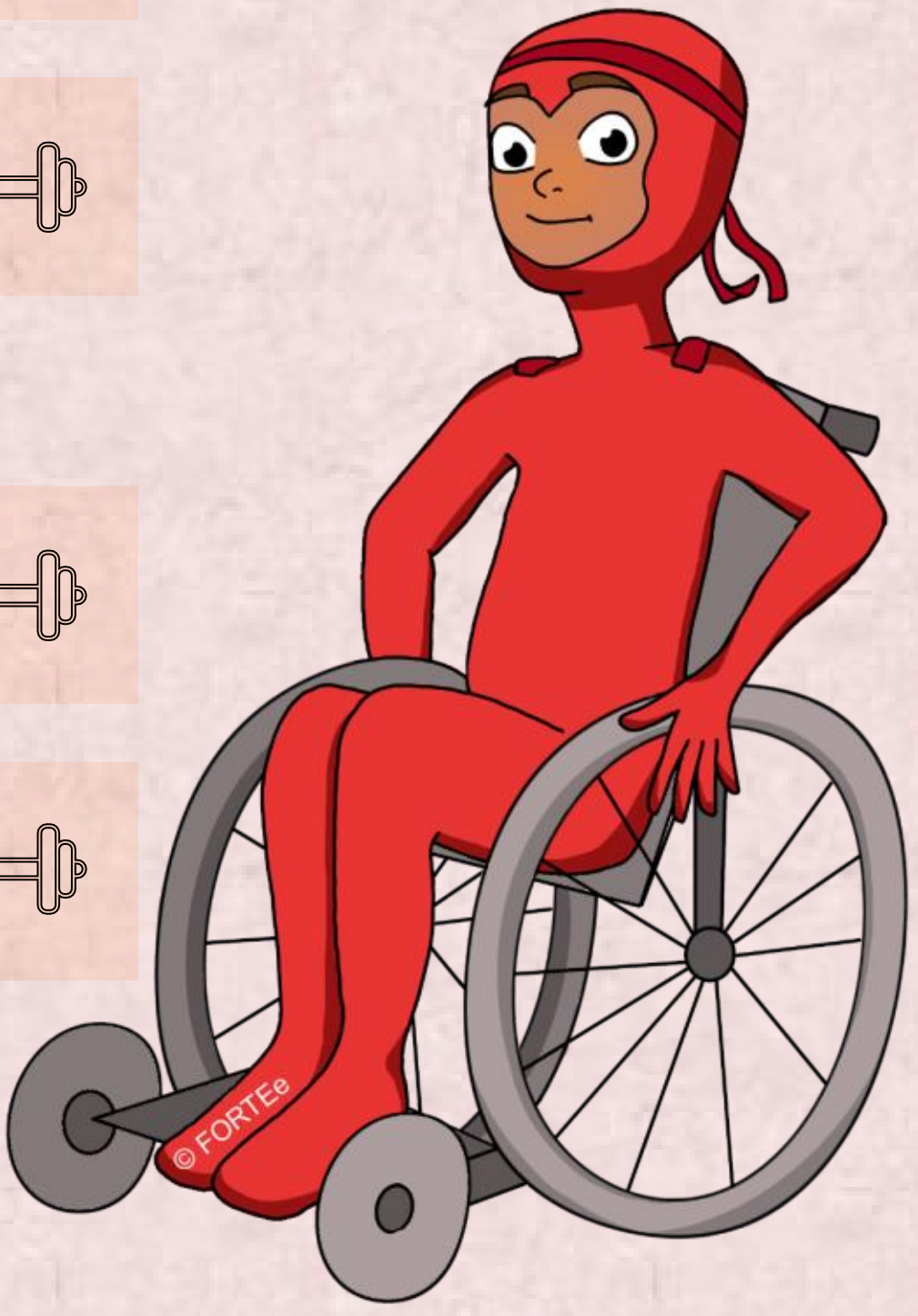

Strength – Lower body

Week 1					
Week 2					
Week 3					
Week 4					
Week 5					
Week 6					
Week 7					
Week 8					
Week 9					
Week 10					

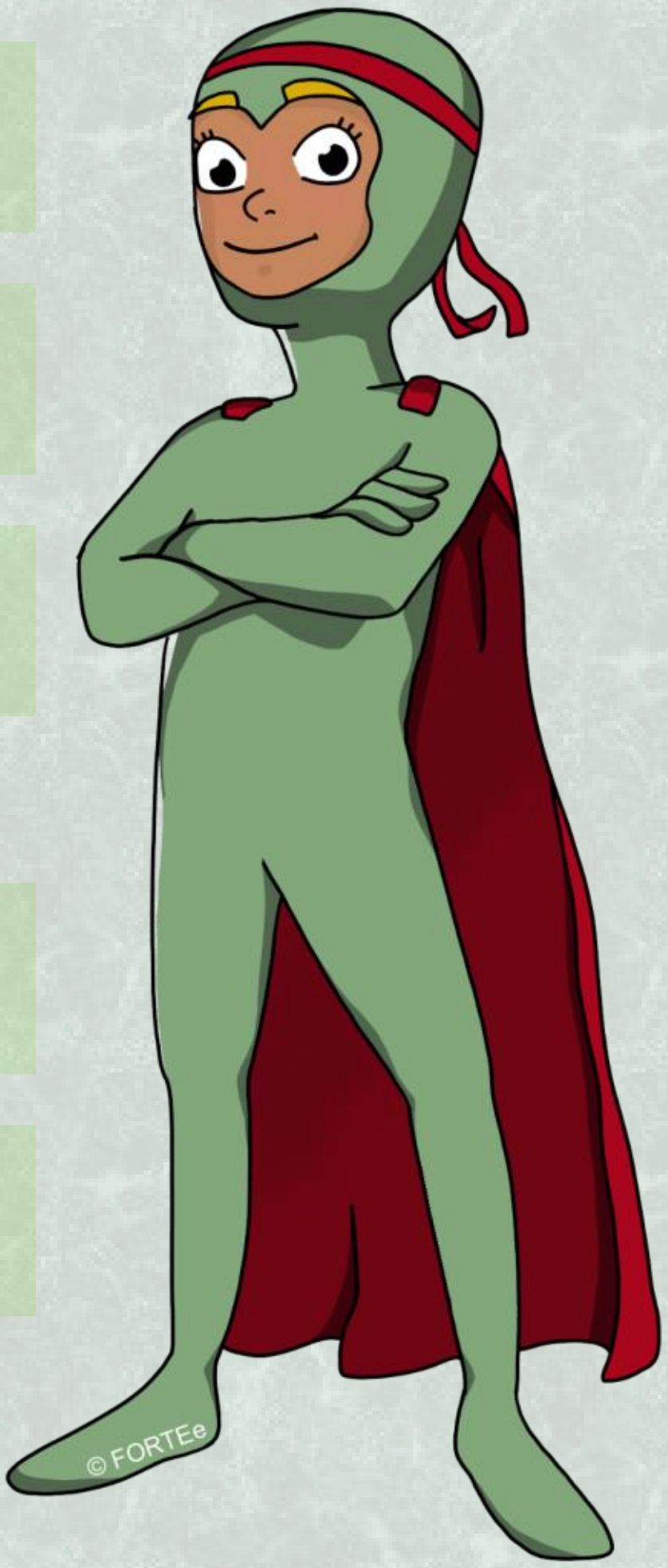

Flexibility

Week 1					
Week 2					
Week 3					
Week 4					
Week 5					
Week 6					
Week 7					
Week 8					
Week 9					
Week 10					

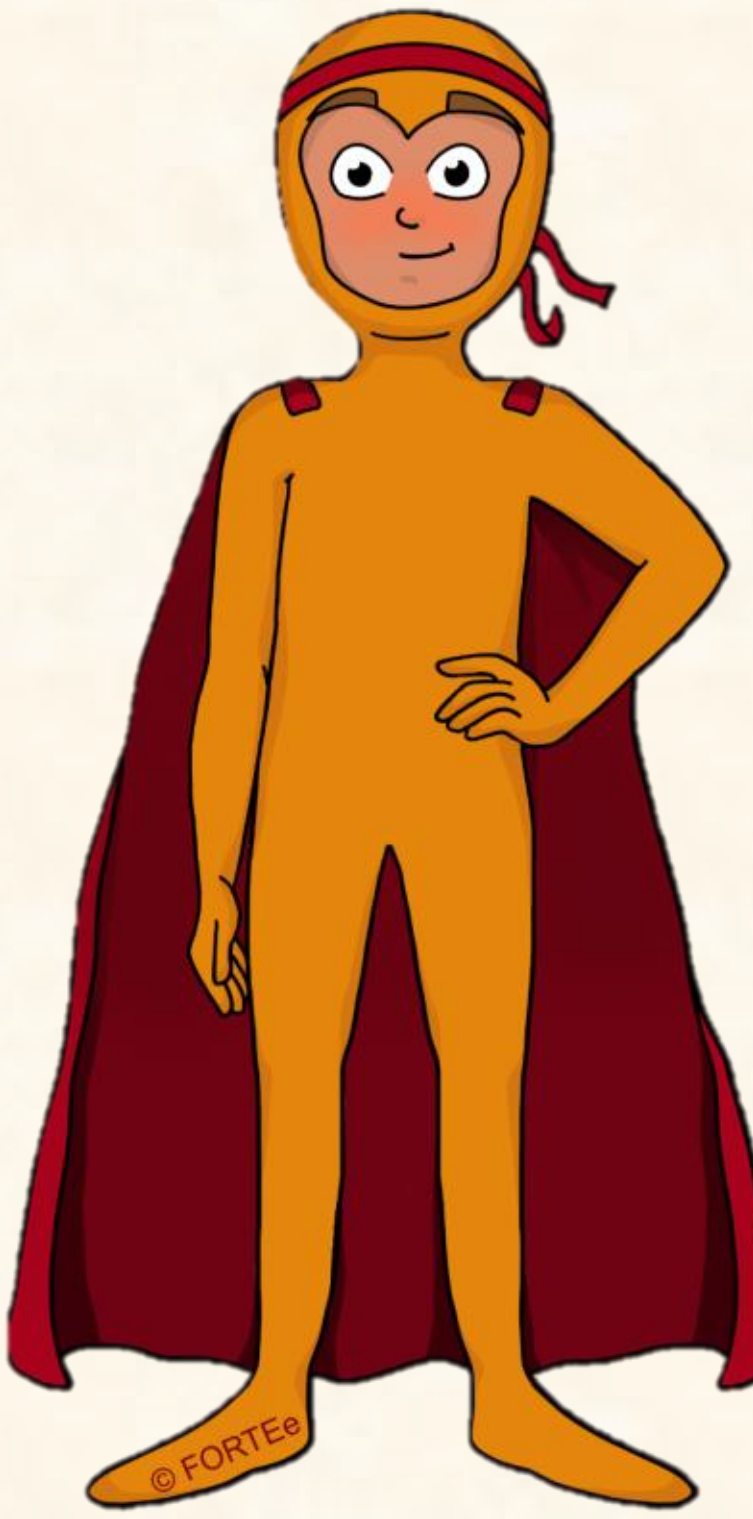

Coordination / Balance

Week 1					
Week 2					
Week 3					
Week 4					
Week 5					
Week 6					
Week 7					
Week 8					
Week 9					
Week 10					

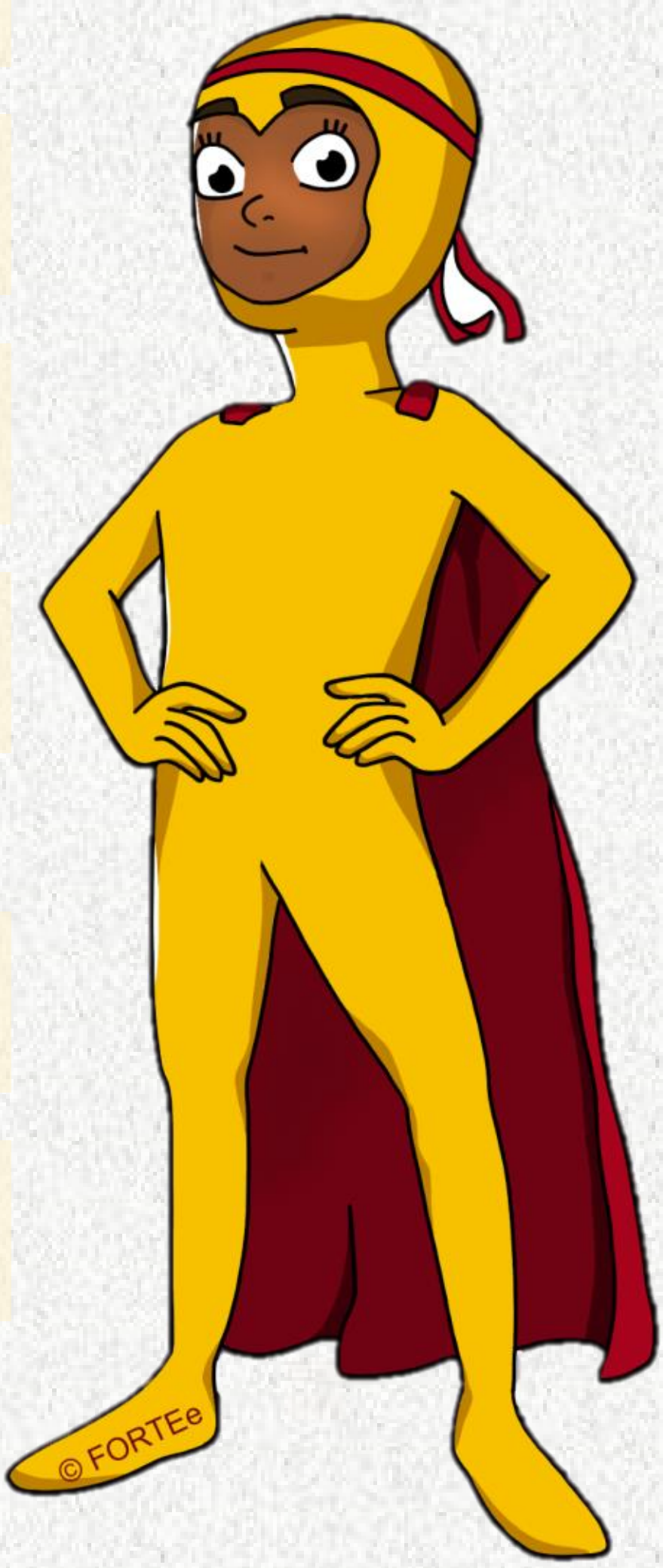

Gait

Week 1					
Week 2					
Week 3					
Week 4					
Week 5					
Week 6					
Week 7					
Week 8					
Week 9					
Week 10					

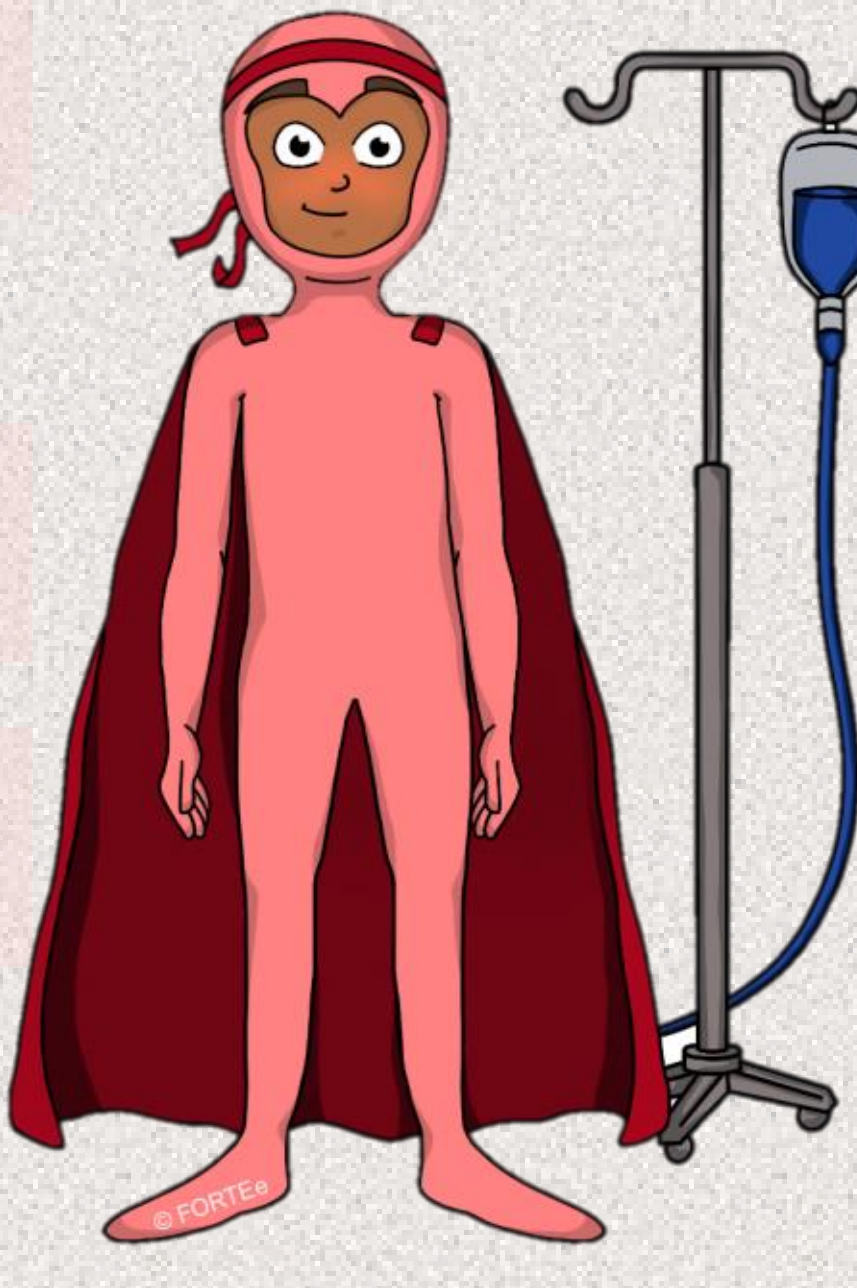

Supplement: Supplementary file 1 [file Datasheet1.pdf]
