# Supplementary material for: Precision-based exercise protocols for children with cancer: a methodological approach from the European FORTEe research project
Source: Front Pediatr. 2026 Sep 17;14:1907775. doi: 10.3389/fped.2026.1907775 (PMC13627370; doi:10.3389/fped.2026.1907775)
Supplement: Supplementary file 2 [file Datasheet2.pdf]

TRAININGS FOR SPECIFIC CLINICAL CONDITIONS

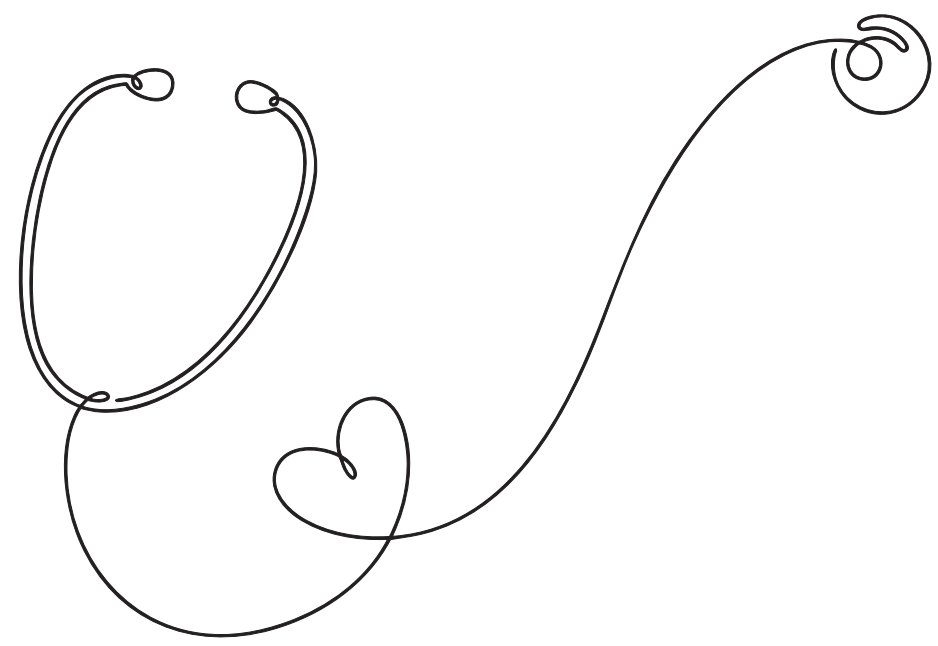

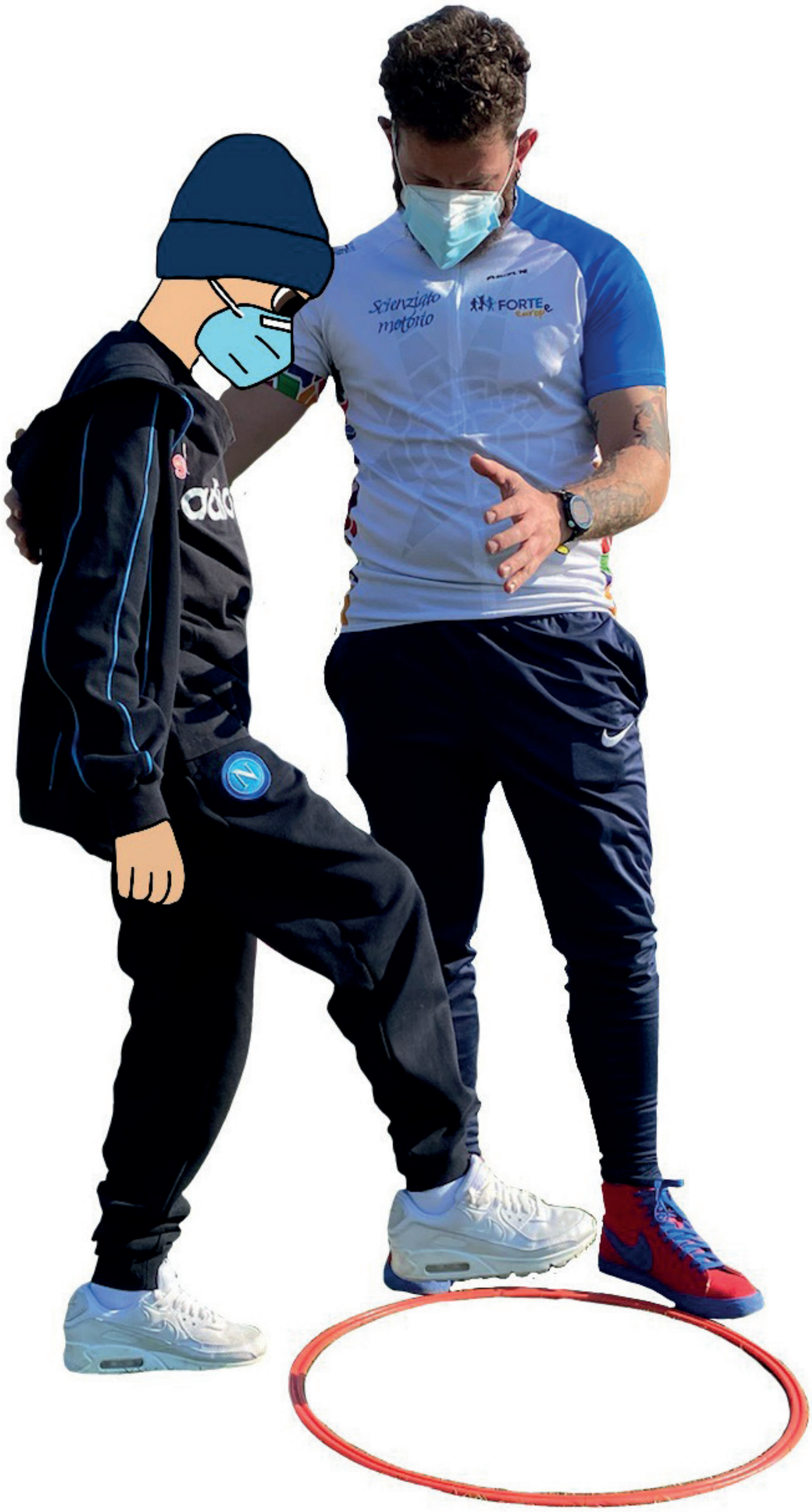
Scienziato motorio
FORTE

THE CLINICAL PERSPECTIVE

When I was first approached with the idea of a structured program of precision-based exercise for children and adolescents with onco-haematological diseases, I must admit that I was skeptic. Us all who work with extremely fragile patients know that their bodies are challenged by intensive chemotherapies and treatment-related complications and their mood is often jeopardized by hospitalization, isolation from peers and fear to fail treatment.

As physicians, our absolute priority has always been to protect our patients. To protect them from infections, complications and any avoidable risk. Even more as pediatricians. In this context, the concept of physical training, even if adapted and carefully supervised, could initially seem like a step too far.

Over time, however, my perspective deeply changed. Based on my daily clinical practice. Day after day, I observed the effects of carefully designed and safely performed tailored exercise, which was progressively implemented into each patient treatment strategy. The improvements involved not only physical abilities (strength, coordination and mobility) but also deeper, equally important aspects, such as mood, self-confidence, body awareness and ability to face the challenges of treatment with greater energy and engagement. What initially appeared to be a potential risk has proven to be a valuable resource, when driven by specific expertise. This is not just about 'doing sports' in its conventional sense, but about supporting children and adolescents through a process which aims in the direction of enhancing the ability to move, in order to restore independence and normality, which are inevitably taken away by the disease.

The FORTEe exercise protocols' book is the result of this realisation, offering a structured, thoughtful and scientifically sound approach to support precision-based exercise in children and adolescents with onco-haematological diseases. The exercises presented here are the result of rigorous work and are designed to respect the clinical limits of patients while enhancing their potential.

I can now claim that precision-based exercise program are an unmissable part of our medical approach. Each child or adolescent is not just his disease, is not just a patient, but is a potential athlete, competing to gain his life back against his disease. Treating a patient as a whole person also means restoring movement, autonomy and hope. Which should always be possible.

Let's take the field. In this kind of sport competition the winner won't be just the best athlete, but them all. Our societies will win, as our cancer survivors will ultimately become adults who have learned how to improve their abilities and respect their competitors.

Adriana Cristina Balduzzi
Head of the Pediatric Haematology-Oncology Unit, Monza

THE FUTURE IS ACCOMPANIED BY ACTS OF CARE

What makes our model of care for pediatric haematological and oncological diseases unique is our belief that every child should be at the centre of their own journey through illness, from diagnosis throughout treatment and beyond. This principle is based on a partnership between families, clinicians, and researchers. The dream of making children feel safe and allowing them to stay in hospital without fear – knowing they would find competence, attention, respect, and total acceptance of their situation – led to the launch of the Sport Therapy project shortly after the Maria Letizia Verga Centre was established.

The idea of doing sport – or rather, devising personalised training programs adapted to each child's clinical condition – really meant 'throwing our hearts over the obstacle', trusting in the dream that started our journey over forty-five years ago and still drives us today when families experience a 'break in normality' due to diagnosis.

Physical exercise is a concrete way of giving children back what illness and therapeutic treatments take away: the strength to move, run and jump, and with it, their thoughts about the future.

What initially seemed like a bold idea has, in recent years, proved to be a valuable resource thanks to a multidisciplinary team of professionals, including pediatric oncologists, sports medicine physicians, exercise professionals, osteopaths, child neuropsychomotor therapists, psychologists and educators. This team has been able to transform physical exercise into a valuable tool to support the treatment process.

At this moment of greatest fragility, when families feel the need to protect their children from every possible risk, physical exercise becomes an integral part of their journey together. It is a personalised intervention that is carefully supervised and adapted to each child's clinical condition. This is only possible thanks to constant and reassuring dialogue with pediatric oncologists, who can help parents to transform fear into confidence. This allows their children and young people to participate in training programs with awareness and serenity.

In the Centre's gym, on the equipped terrace, and even in hospital rooms when clinical conditions prevent patients from leaving bed, our exercise professionals provide daily support tailored to each child's or young person's clinical needs and capabilities. This training practice generates not only psychological and physical well-being, but also becomes a metaphor for facing the challenge of illness. It teaches individuals to think of themselves as capable of enduring pain and fear, and above all, it shows them that they are not alone in facing illness.

THE FUTURE IS ACCOMPANIED BY ACTS OF CARE

This is why the FORTEe project confirms to us that all of this is possible and can become standard practice. The exercise protocol manual is a valuable scientific achievement and a concrete sign of what can happen when clinicians, researchers and parents share the same vision: that even during illness, it is important to continue caring for each child's life project and safeguarding their desire for the future.

Mariapaola Verga
Vice-President of the Maria Letizia Verga Foundation, for the study and treatment of childhood leukaemia

WHEN THE RESPIRATORY SYSTEM IS NOT EFFICIENT

WHY?

Acute conditions: infection, fluid overload, cytokine release syndrome, acute pulmonary graft-versus-host disease.
Chronic conditions: chronic pulmonary graft-versus-host disease.

OUR FEAR

Hypoxia.
Fatigue.

ADAPTATION

Small skeletal muscle groups exercises are allowed in all conditions. Moderate cardiopulmonary exercise allowed in chronic conditions.

RECOMMENDED

Use the pulse oximeter during the training.
Seated position.
Do not lie totally on your back for more than 2 minutes at a time.

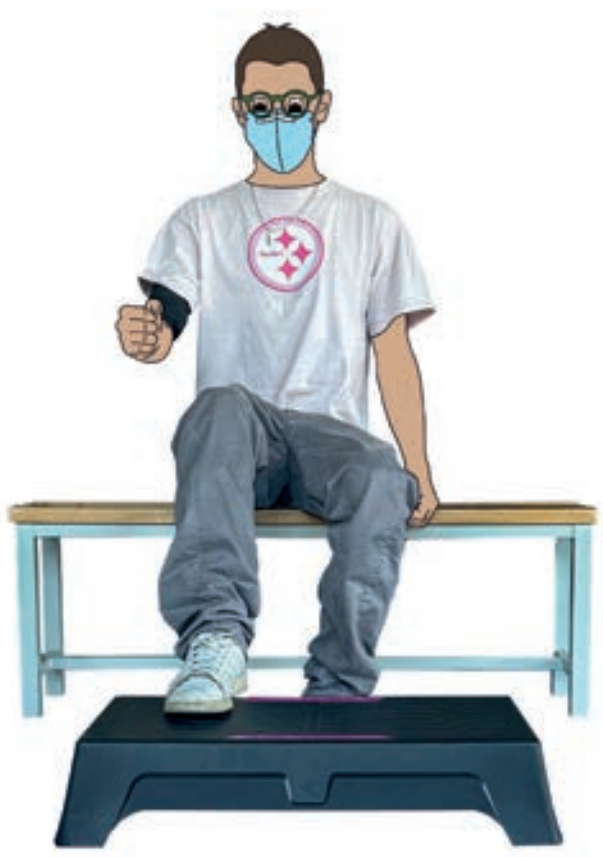

Step up, eyes open, alternative
20 sec exercise and 40 sec recovery, in total 5 min
on p. 77

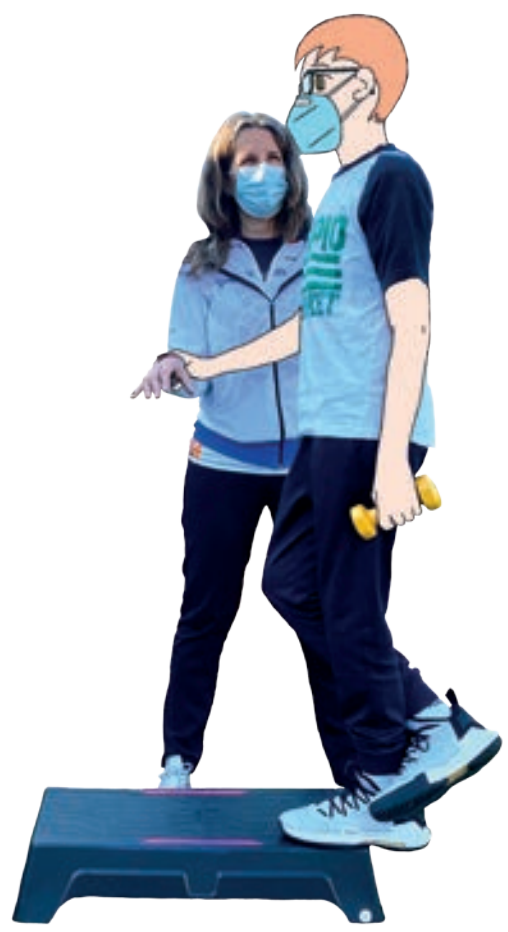

Calf raise
6-8 reps for 3 sets
on p. 120

WHEN THE RESPIRATORY SYSTEM IS NOT EFFICIENT

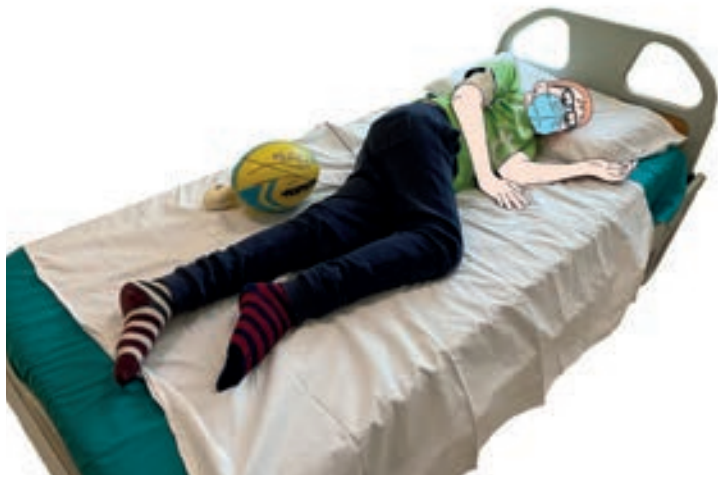

Adduction
6-8 reps for 3 sets
on p. 124

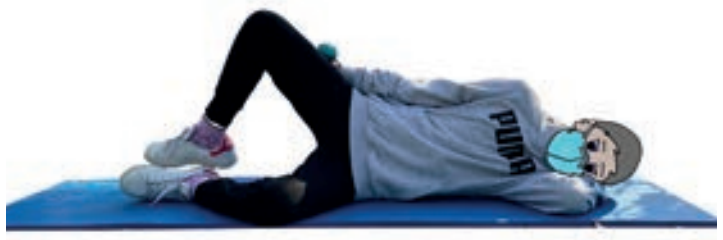

Clamshell
6-8 reps for 3 sets
on p. 126

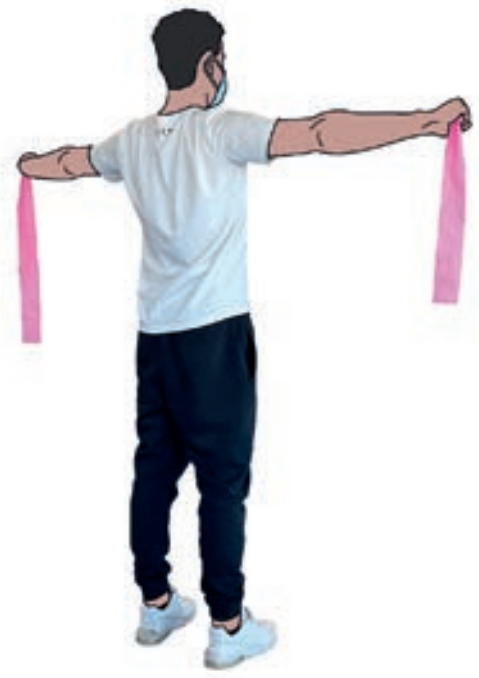

Elastic band rear raise
6-8 reps for 3 sets
on p. 154

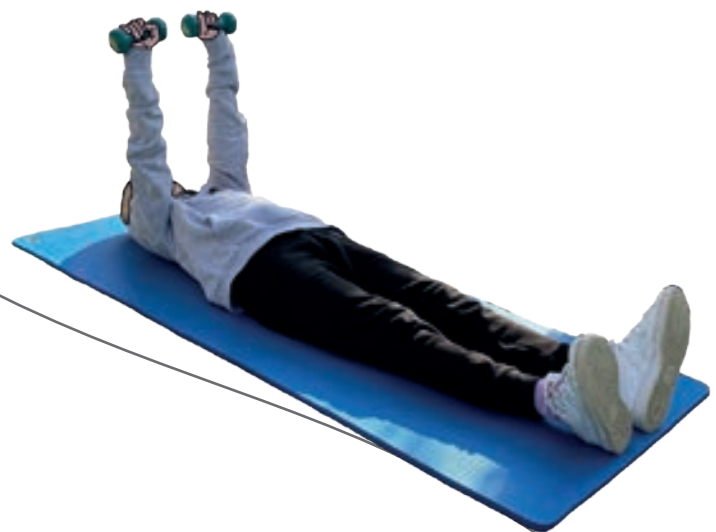

Bench press
6-8 reps for 3 sets
on p. 160

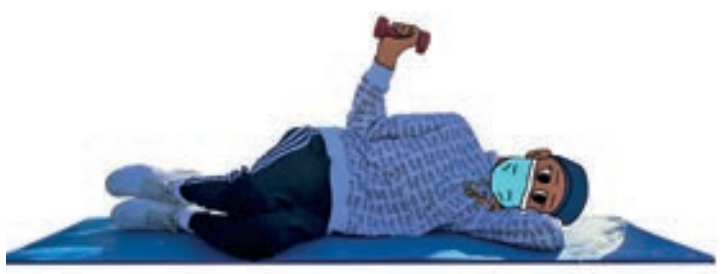

Shoulder external rotation
6-8 reps for 3 sets
on p. 162

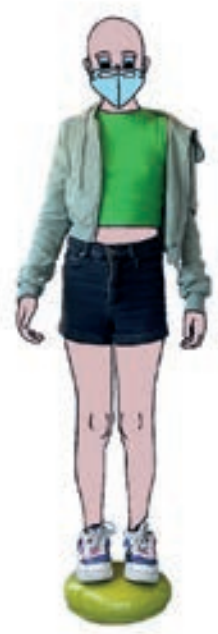

Bipedal posture on balance disc
10 sec exercise for 4 sets
on p. 218

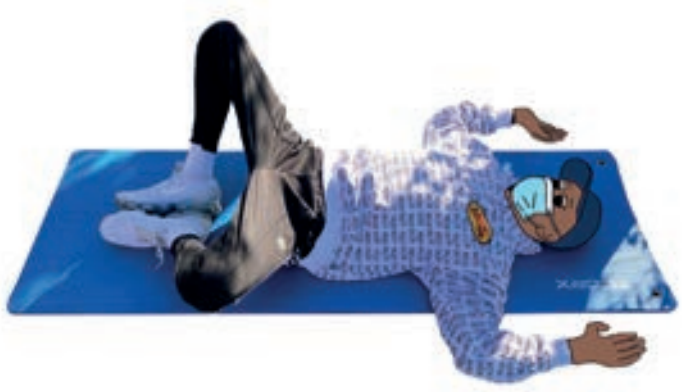

Chandelier
45 sec
on p. 250

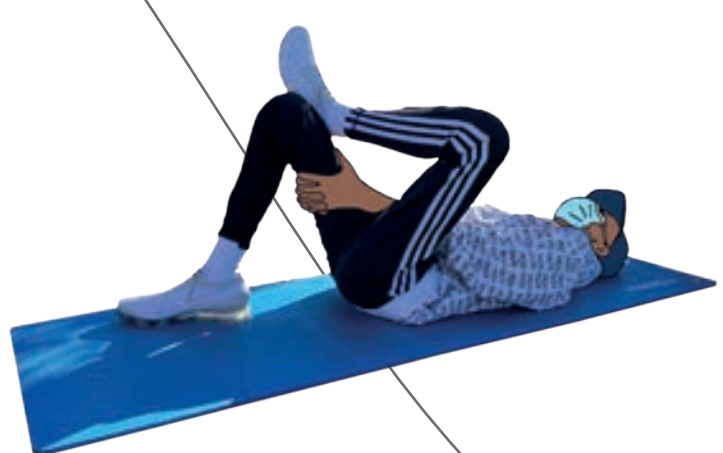

Padlock
30 sec each leg
on p. 252

WHEN THE CARDIAC OUTPUT IS NOT EFFICIENT

WHY?

Acute conditions: infections, sepsis, anthracycline damage, fluid overload, cytokine release syndrome.

OUR FEAR

Hypoxia.
Hypotension.
Fatigue.
Heart failure.

ADAPTATION

Small skeletal muscle groups exercises are allowed in all conditions. Moderate cardiopulmonary exercise allowed in heart failure.

RECOMMENDED

Use the pulse oximeter during the training.
Seated position.

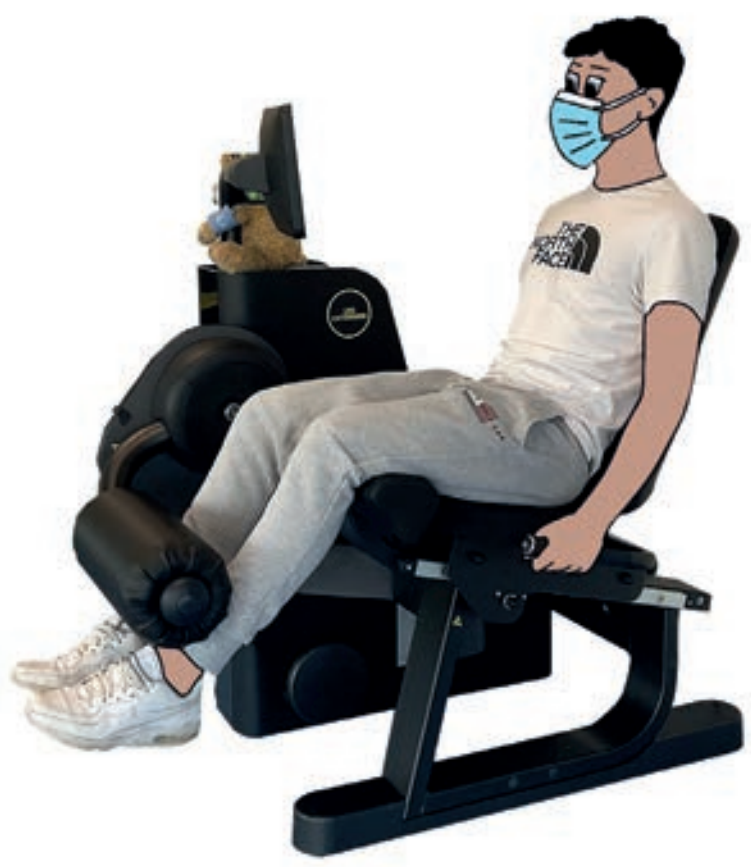

Leg extension
6-8 reps for 3 sets
on p. 132

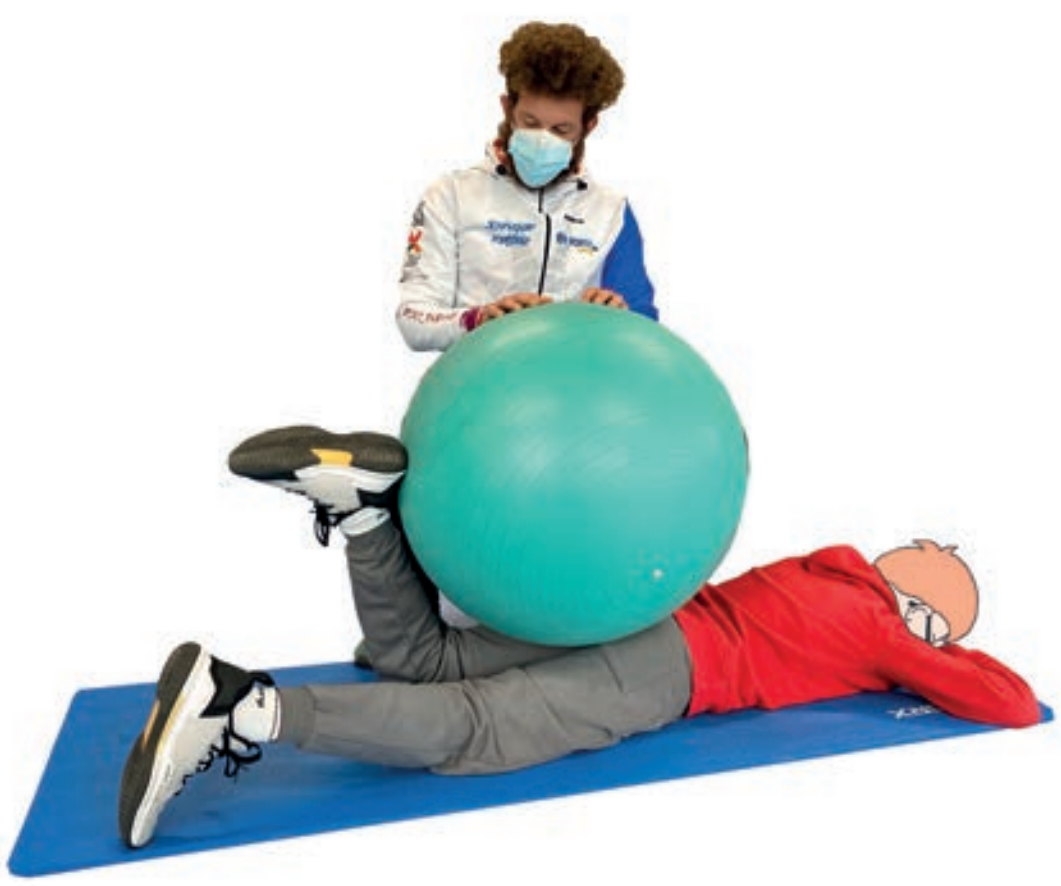

Leg curl
6-8 reps for 3 sets
on p. 134

WHEN THE CARDIAC OUTPUT IS NOT EFFICIENT

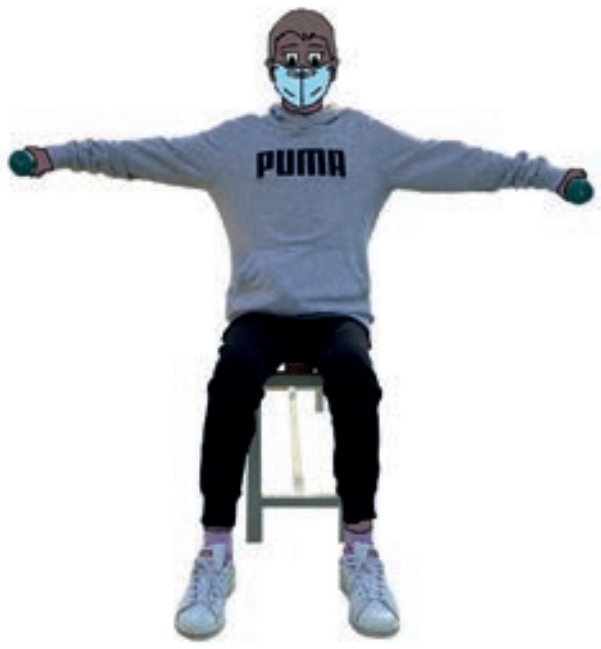

Lateral raise
6-8 reps for 3 sets
on p. 174

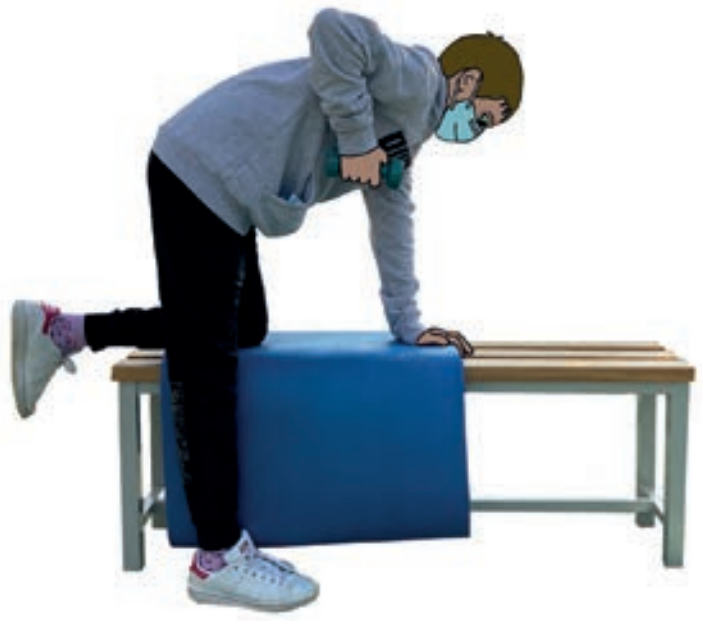

One arm dumbbell row
6-8 reps for 3 sets
on p. 144

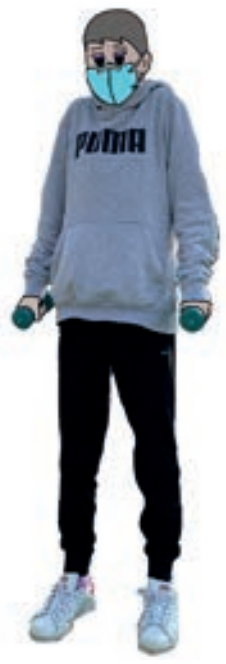

Dumbbell shrug
6-8 reps for 3 sets
on p. 166

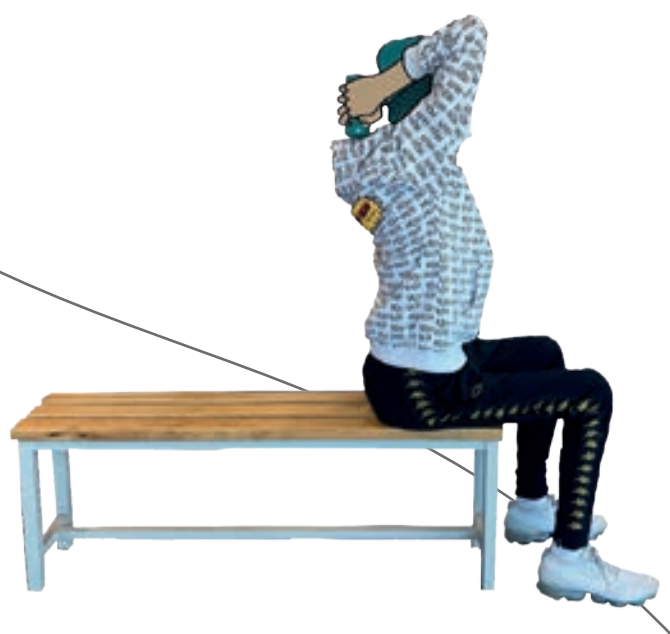

Triceps extension
6-8 reps for 3 sets
on p. 177

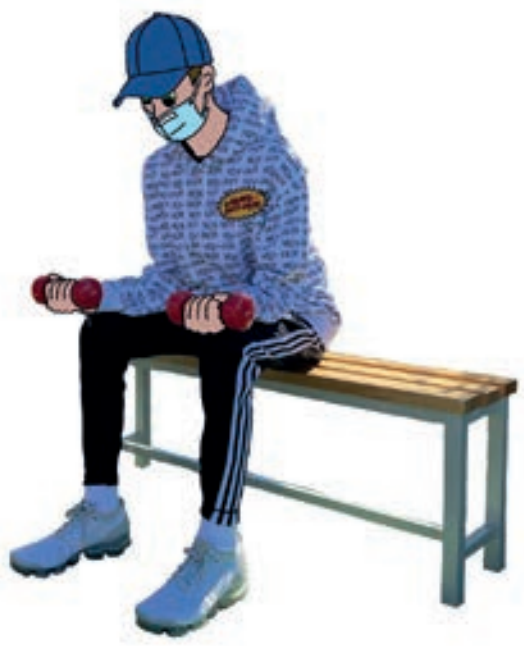

Wrist curl
6-8 reps for 3 sets
on p. 184

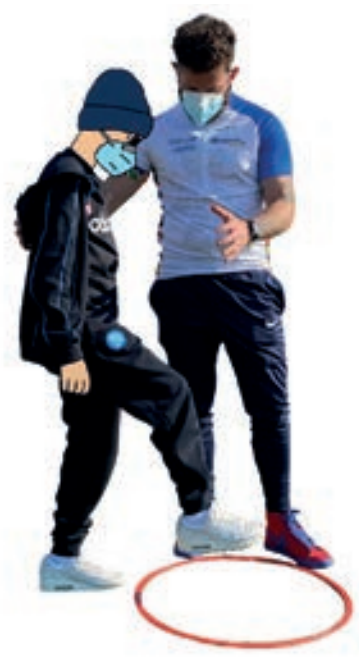

Stepping over obstacles version alternative
10 sec of exercise - repeat again after 30 sec of recovery for 3 sets
on p. 243

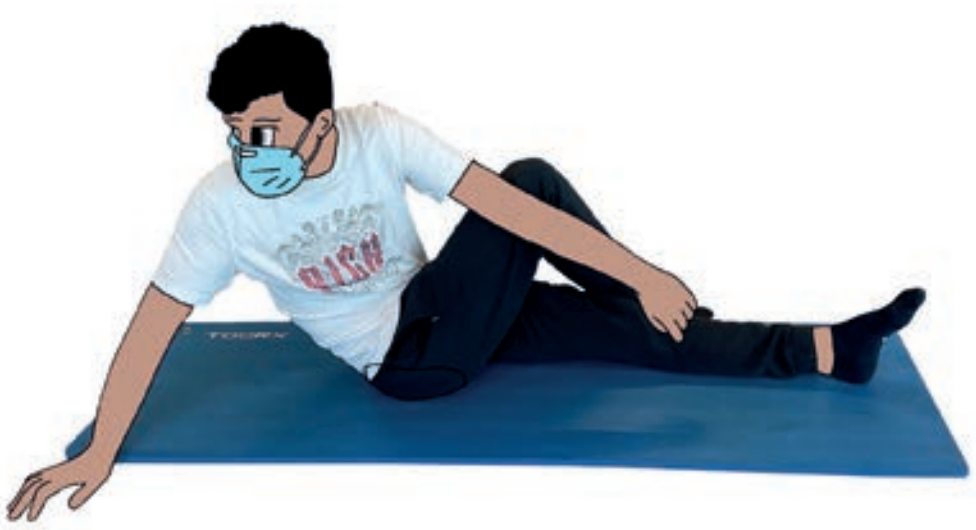

Scorpion
30 sec each leg
on p. 260

OSTEONECROSIS

WHY?

Chemotherapy
side effects.

OUR FEAR

Joint pain.
Risk of fracture.
Exercise makes pain worse.
Accidental falls.

ADAPTATION

Partially loading exercise.

RECOMMENDED

Use the pulse oximeter during the training.
Seated position.

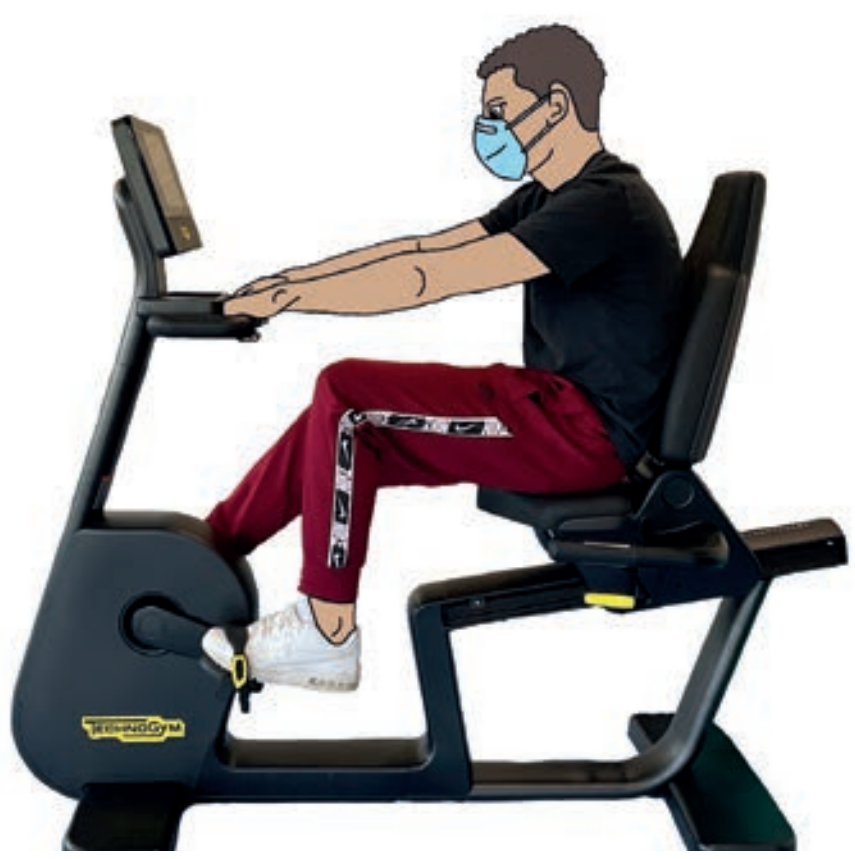

Recumbent bike
6 min of exercise - repeat again after
3 min of rest
on p. 110

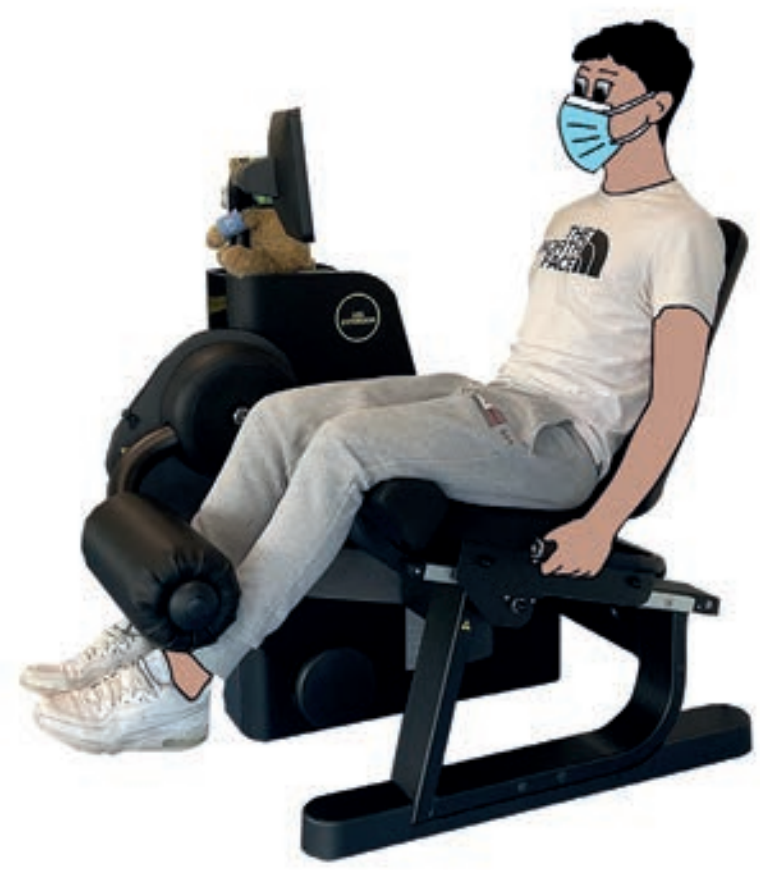

Leg extension
8 reps for 3 sets
on p. 132

OSTEONECROSIS

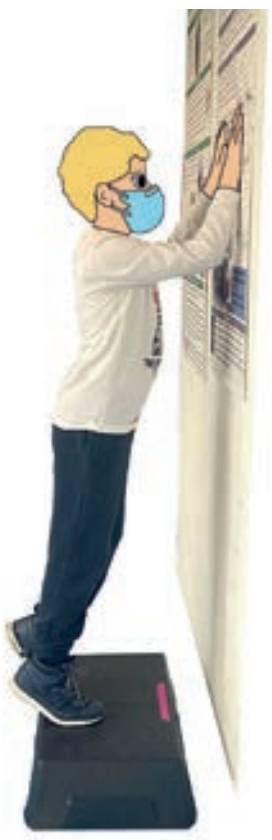

Calf raise from sitting position
8 reps for 3 sets
on p. 121

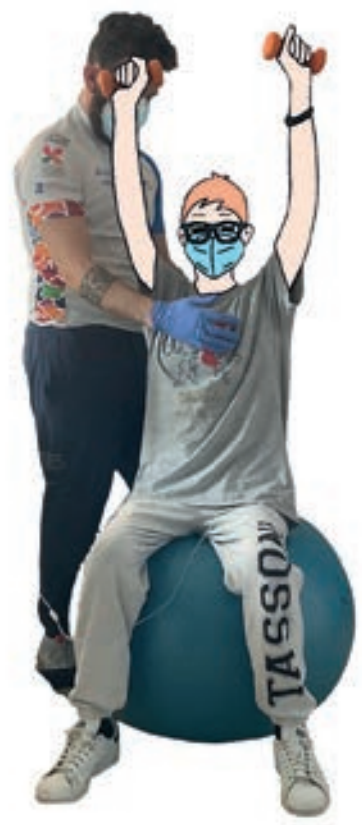

Overhead press
8 reps for 3 sets
on p. 168

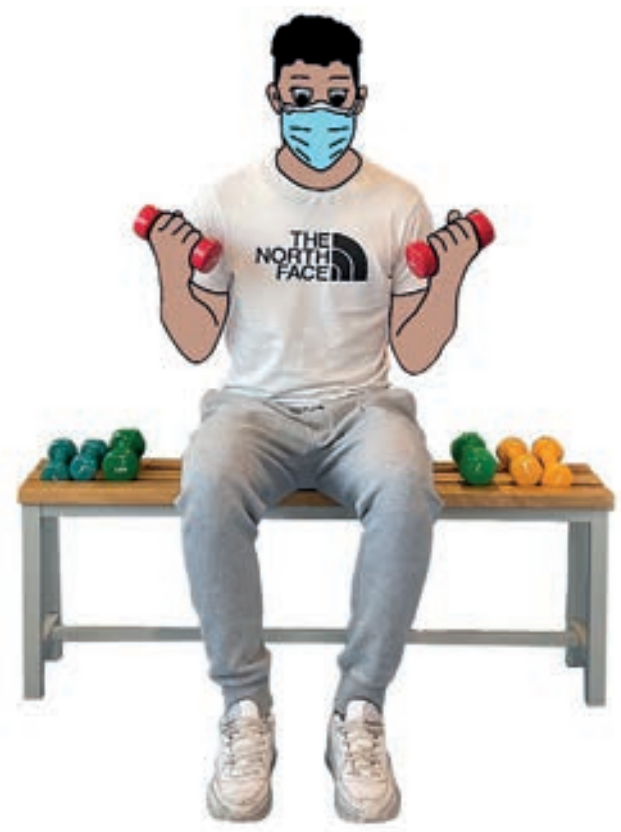

Bicep curl
8 reps for 3 sets
on p. 180

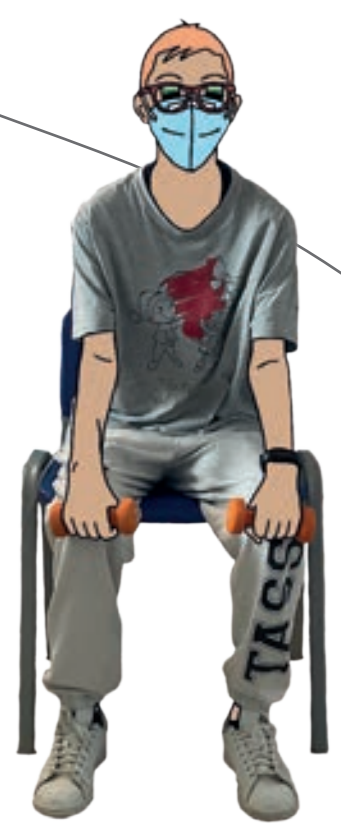

Wrist curl modification
8 reps for 3 sets
on p. 186

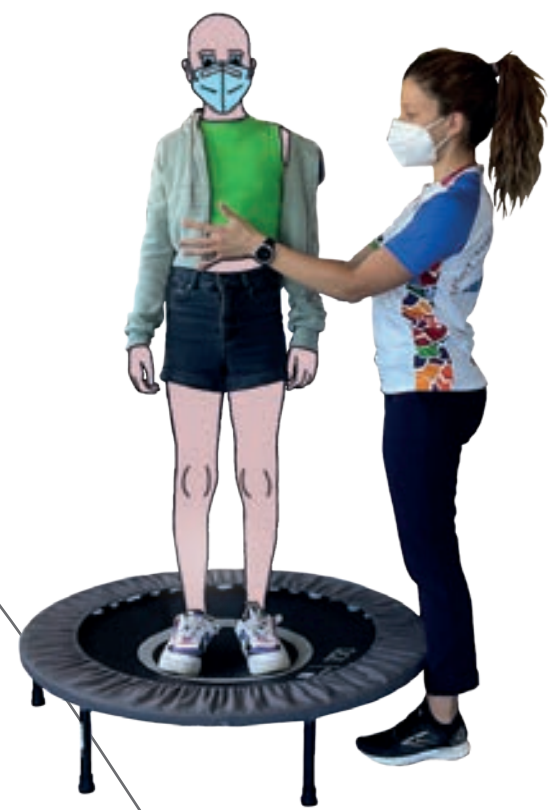

Bipedal posture with eyes open on trampoline, with direct support from the EP
10 sec of exercise - repeat again after 30 sec of recovery for 4 sets
on p. 224

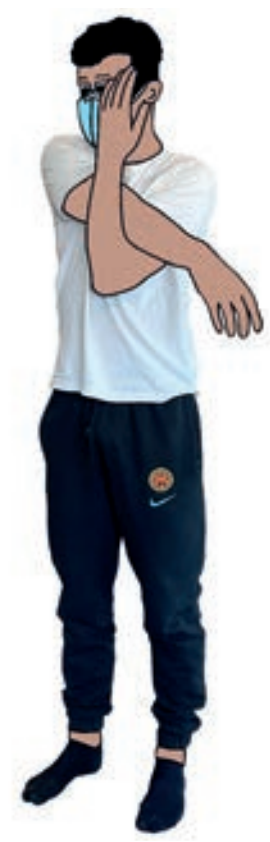

Police man
30 sec for each arm
on p. 264

NEUROMUSCOLAR DISEASES

WHY?

Chemotherapy-induced toxicity, traumatic lumbar puncture, CNS relapse, localised vertebral disease.

OUR FEAR

Accidental falls.
Pain.

ADAPTATION

No monopodalic exercises, partially loading.

RECOMMENDED

Use the pulse oximeter during the training.

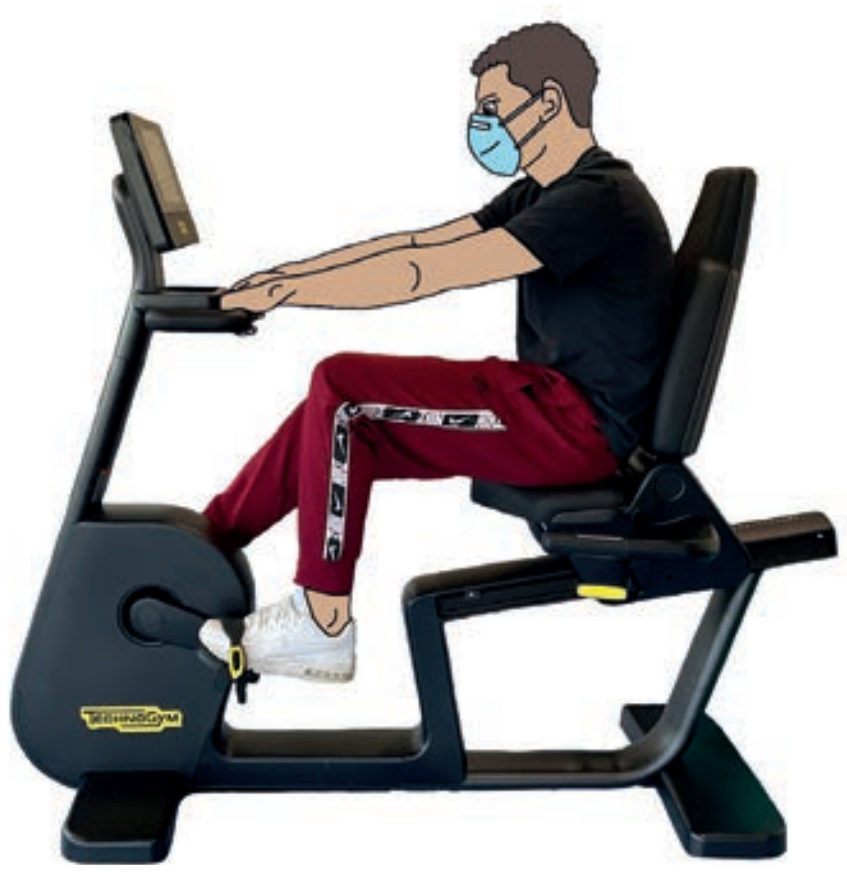

Recumbent bike
3 min of exercise - repeat again after 3 min of recovery for 3 sets
on p. 110

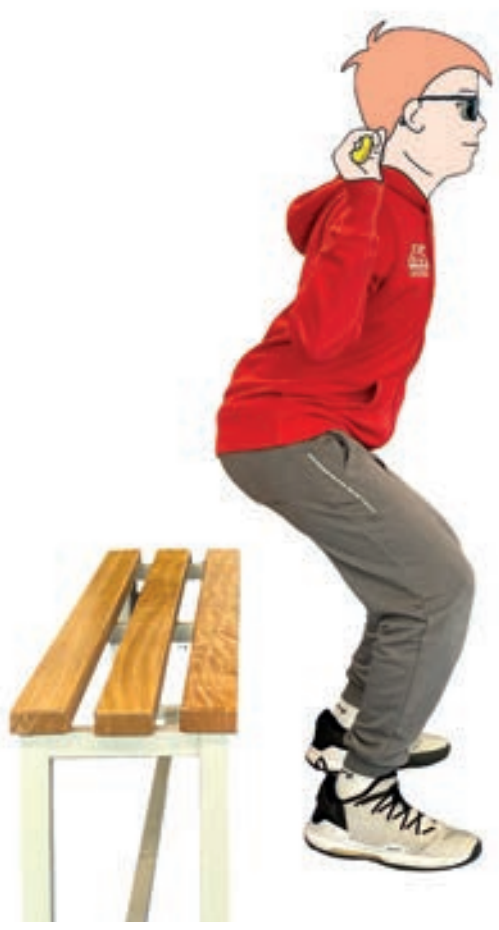

Barbell squat alternative
6-8 reps for 3 sets
on p. 113

NEUROMUSCOLAR DISEASES

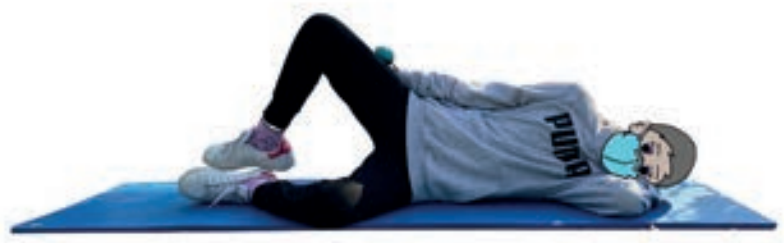

Clamshell
6-8 reps for 3 sets
on p. 126

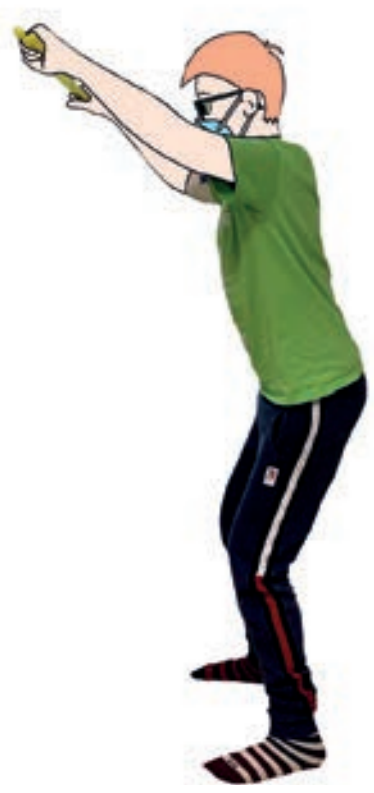

Straight arm pulldown alternative
6-8 reps for 3 sets
on p. 153

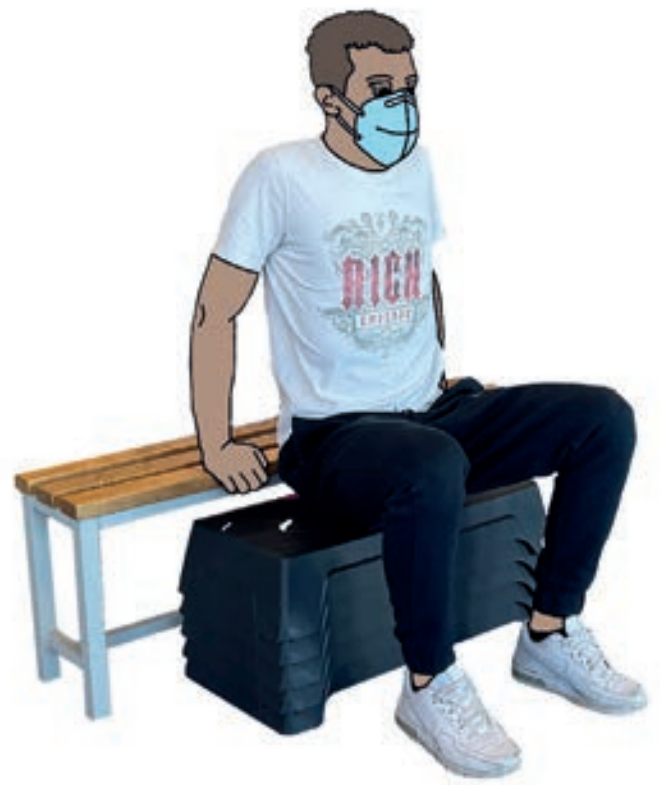

Bench dip alternative
6-8 reps for 3 sets
on p. 179

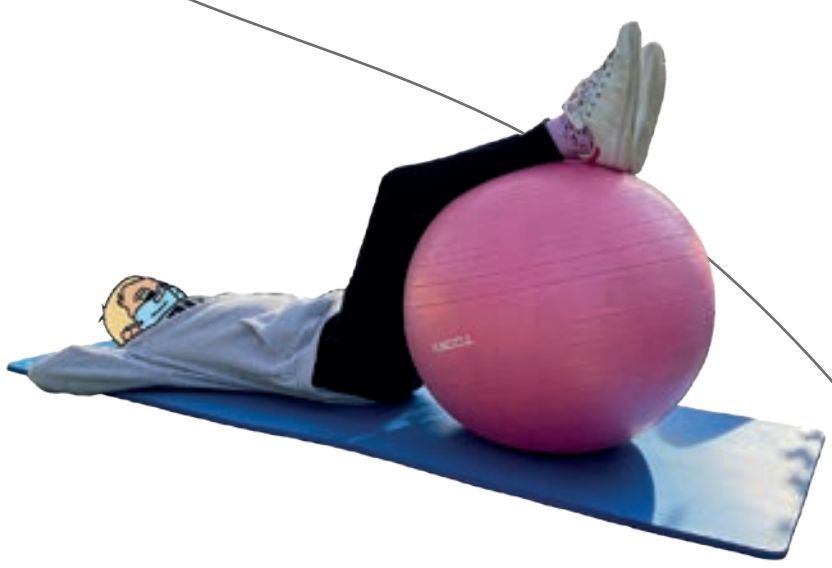

Crunch feet on fiball
6-8 reps for 3 sets
on p. 190

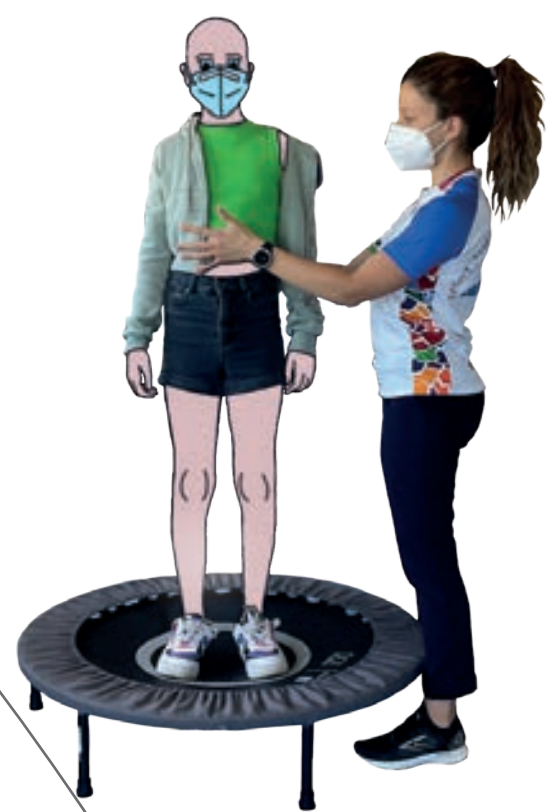

Bipedal posture with eyes open on trampoline
10 sec of exercise - repeat again after 30 sec of recovery for 3 sets
on p. 234

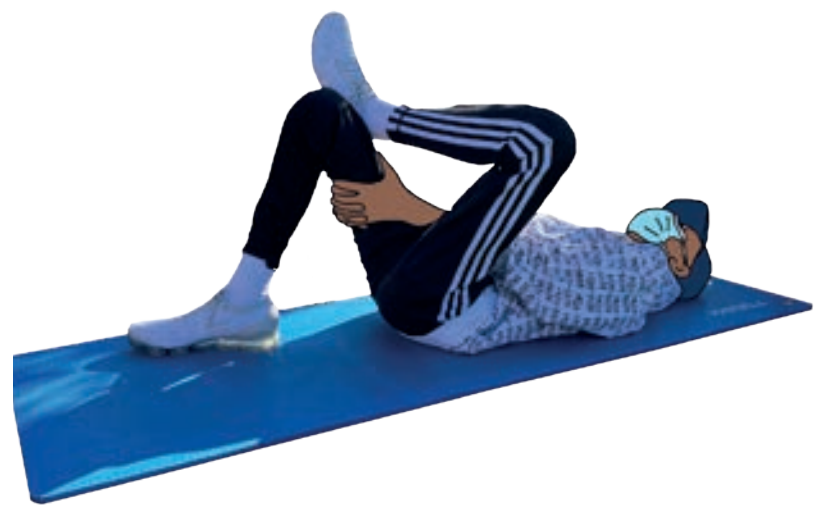

Padlock
30 sec of exercise for each leg
on p. 252

LOW OXYGEN SATURATION

WHY?

Acute or chronic anemia due to myelotoxic chemotherapy, infections, hemoglobinopathies.

OUR FEAR

Hypoxia.
Hypotension.
Fatigue.
Cardiac failure.

ADAPTATION

Intense exercise is allowed but for a few seconds (alactacide metabolism).
Mild cardiopulmonary exercise is allowed.

RECOMMENDED

Use the pulse oximeter during the training.

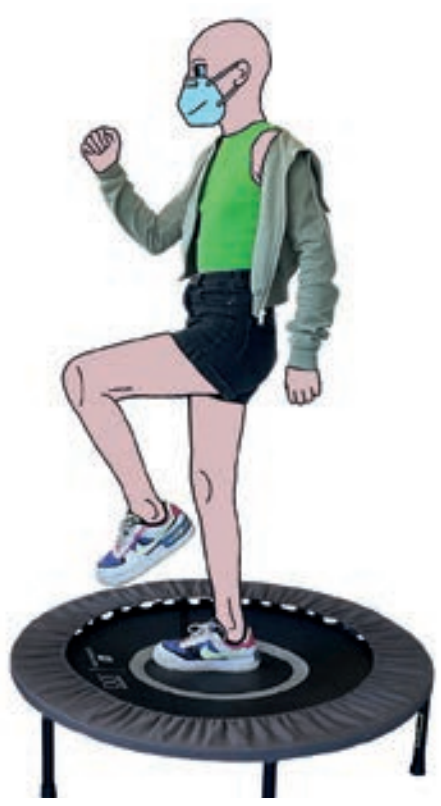

Mini trampoline walk
20 sec of exercise and 40 sec of recovery for 5 min - repeat again after 5 min rest
on p. 68

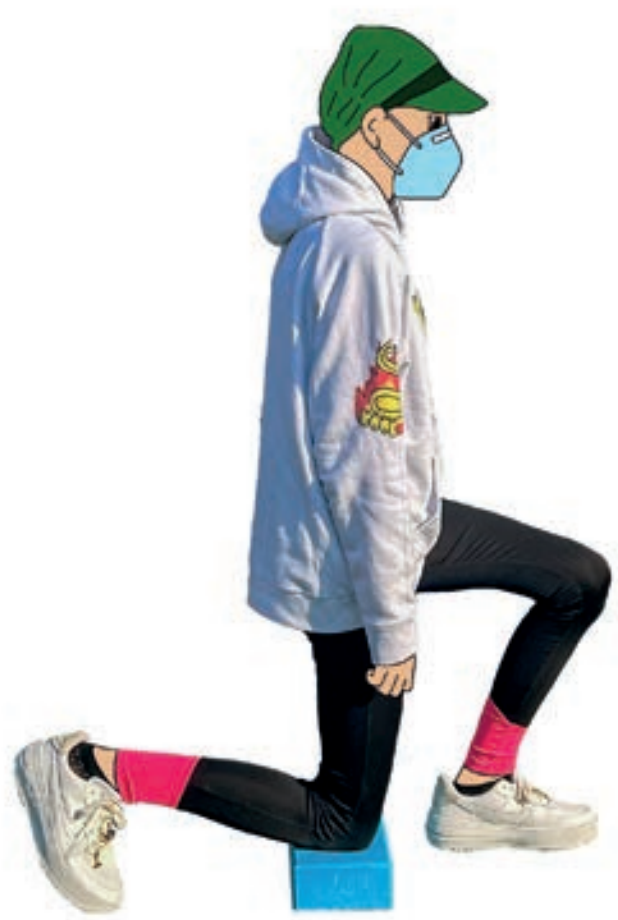

Lunge
6-8 reps for 3 sets
on p. 114

LOW OXYGEN SATURATION

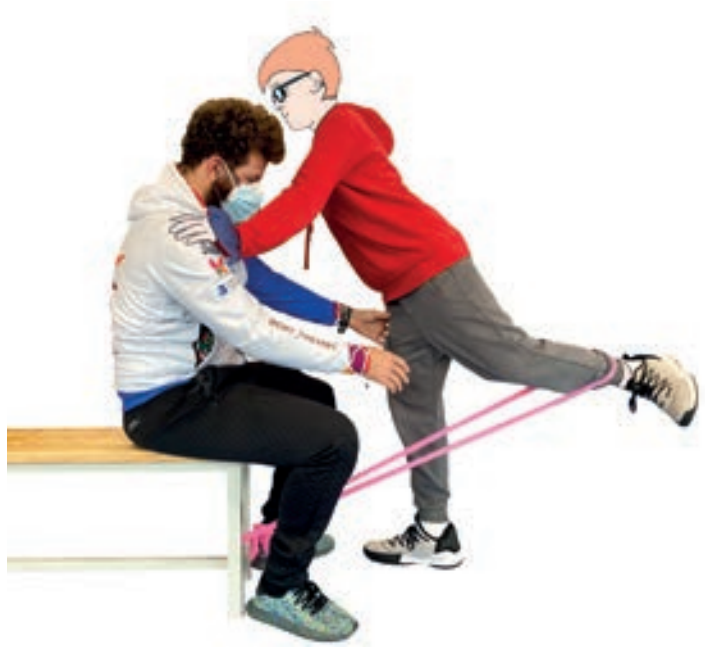

The skater
6-8 reps for 3 sets
on p. 138

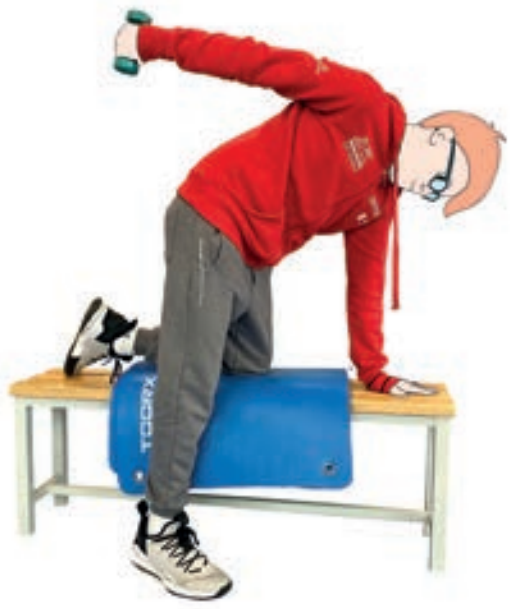

Dumbbell kickback
6-8 reps for 3 sets
on p. 188

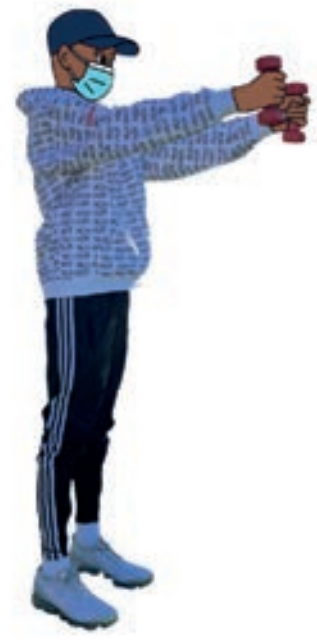

Front raise
6-8 reps for 3 sets
on p. 170

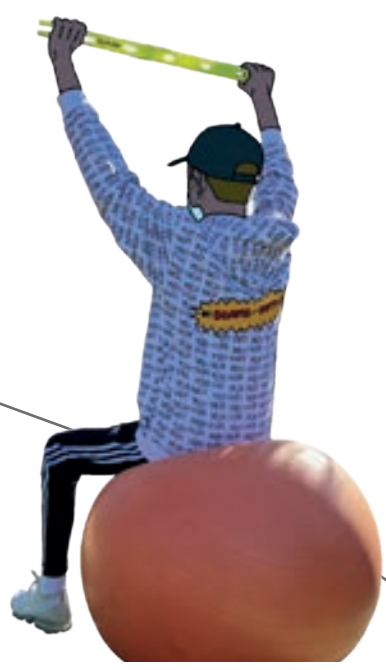

Lat pulldown on fitball
6-8 reps for 3 sets
on p. 150

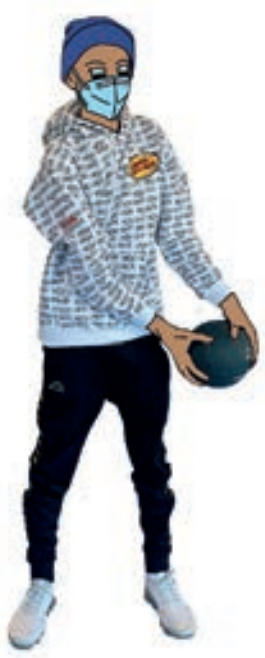

Woodchopper
6-8 reps for 3 sets
on p. 208

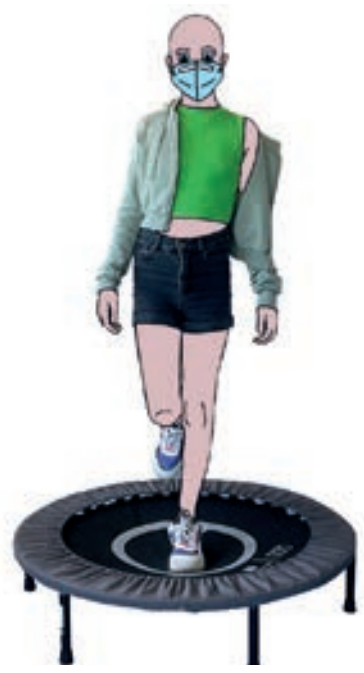

One leg posture with eyes open on trampoline
10 sec of exercise - repeat again after 30 sec of recovery for 4 sets
on p. 224

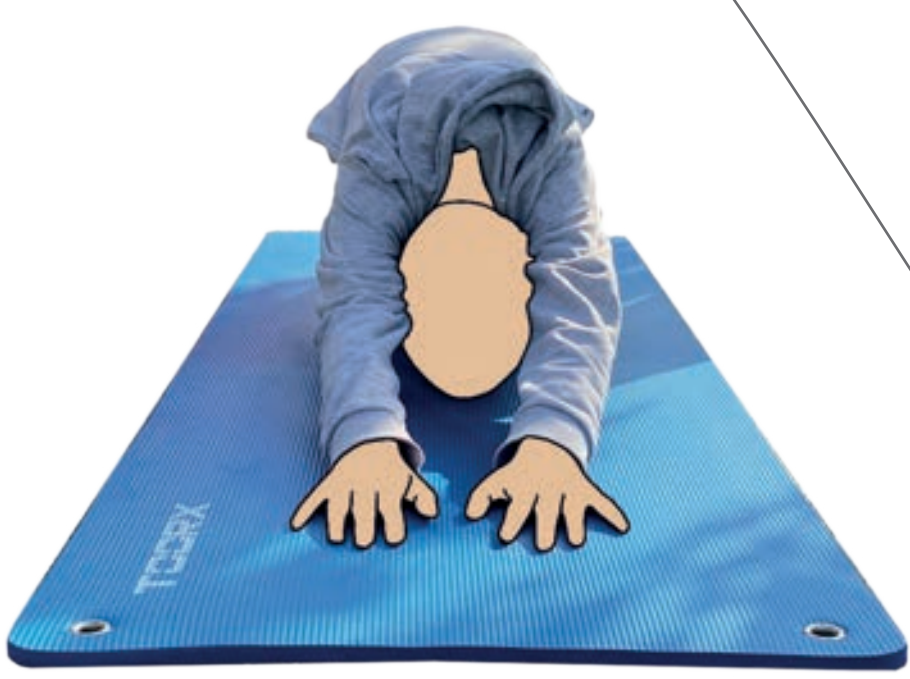

Prayer
40 sec
on p. 256
