# Supplementary material for: Precision-based exercise protocols for children with cancer: a methodological approach from the European FORTEe research project
Source: Front Pediatr. 2026 Sep 17;14:1907775. doi: 10.3389/fped.2026.1907775 (PMC13627370; doi:10.3389/fped.2026.1907775)
Supplement: Supplementary file 3 [file Datasheet3.pdf]

STEP UP, EYES CLOSED, RIGHT LEG UP FIRST

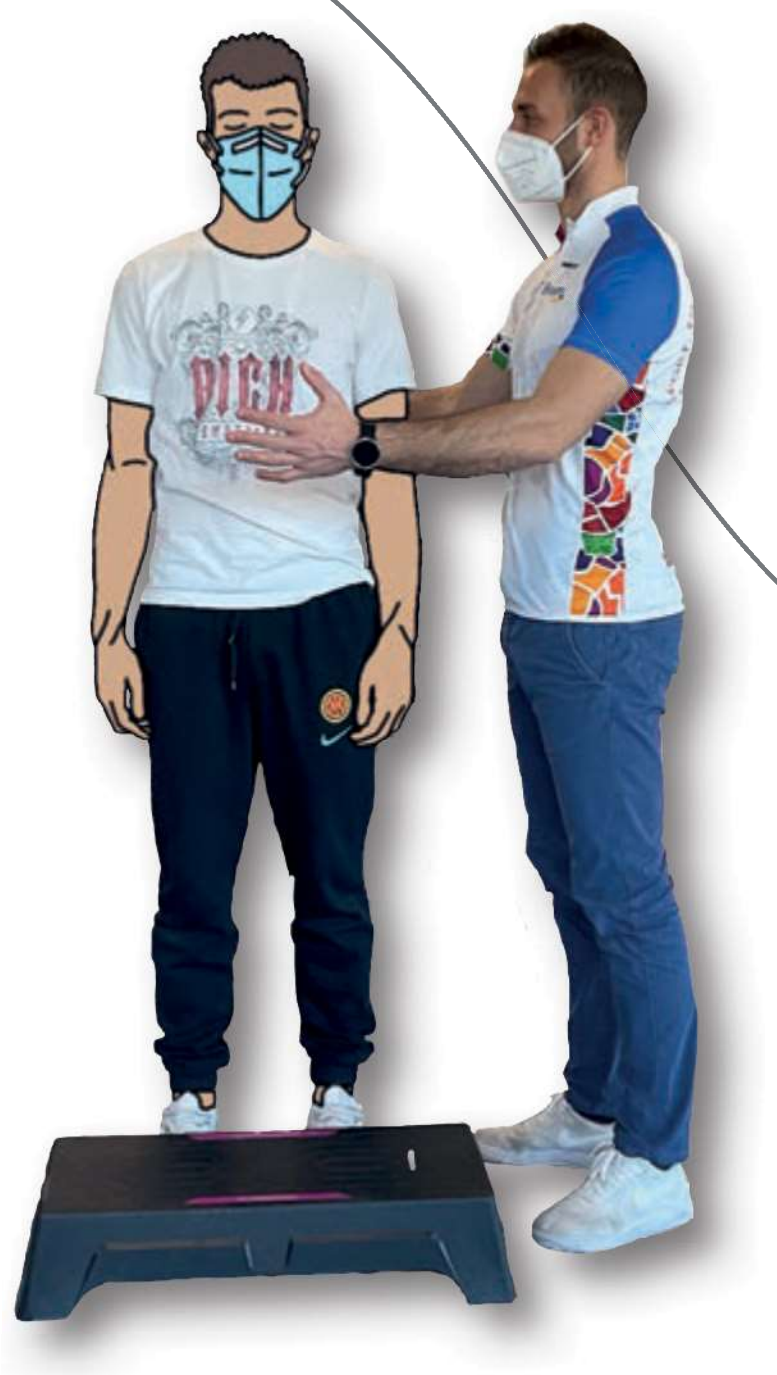

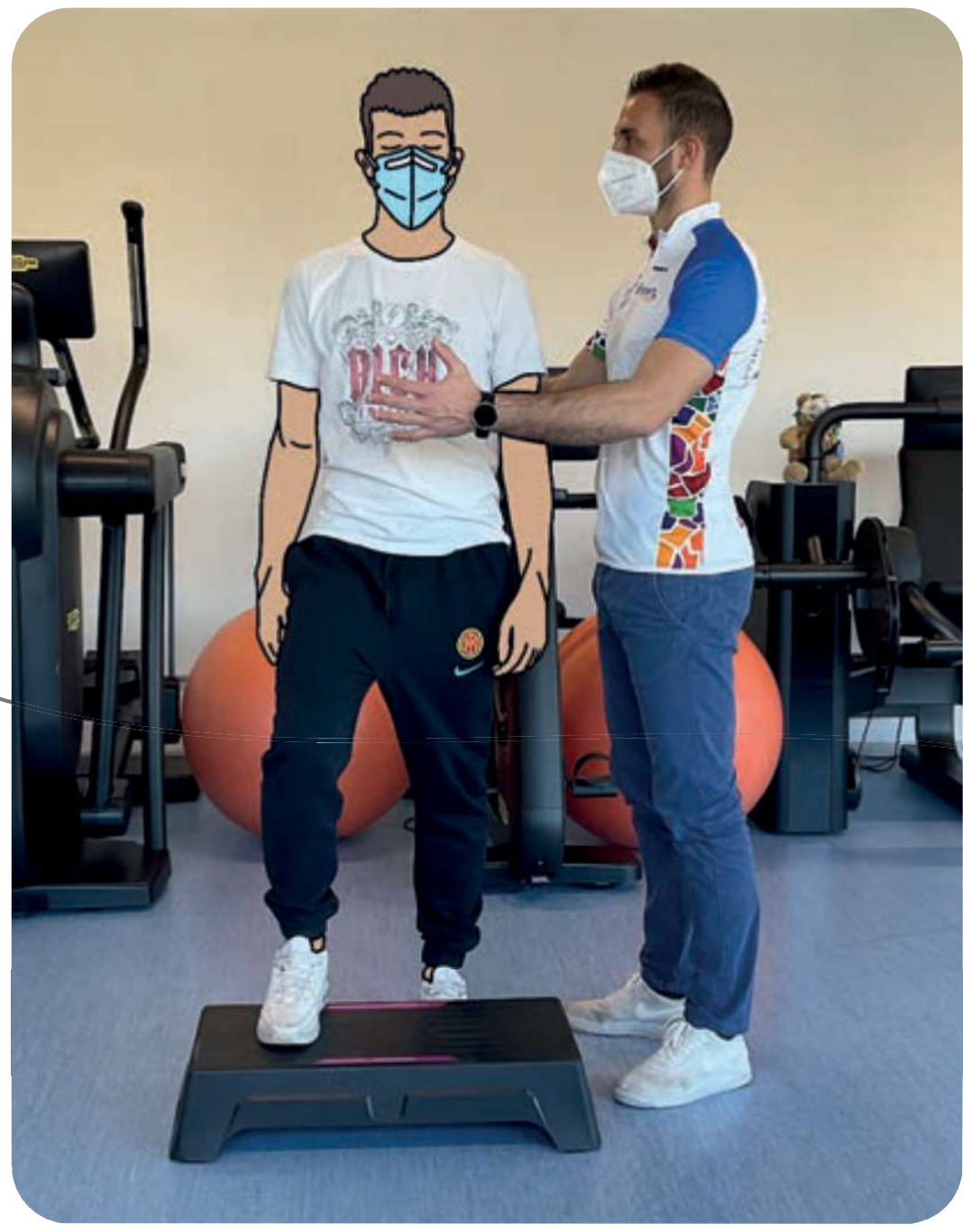

STARTING POSITION

Standing in front of the step with the help of an exercise professional.

ACTION

Step up onto the step keeping your eyes closed. Step up and down with your right leg first, with the help of an exercise professional.

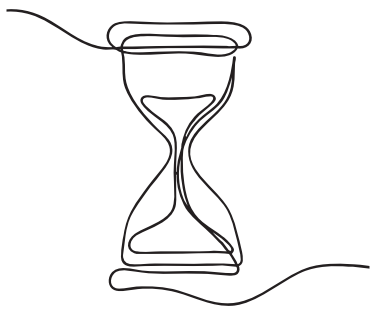

30-60 sec exercise;
30-60 sec recovery;
total time from 2 to 20 min.

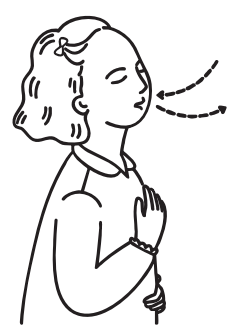

Exhaling as you step up.

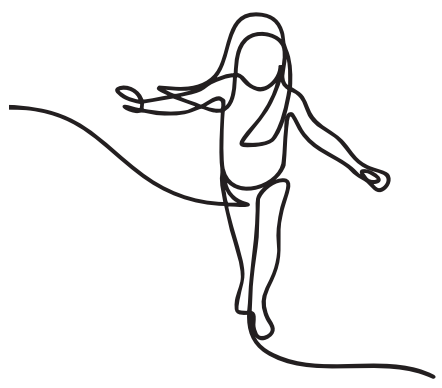

Stabilise by:

- keep your spine in a neutral position;
- keep your chest high and keep your core strong.

STEP UP, EYES CLOSED, RIGHT LEG UP FIRST / ALTERNATIVE

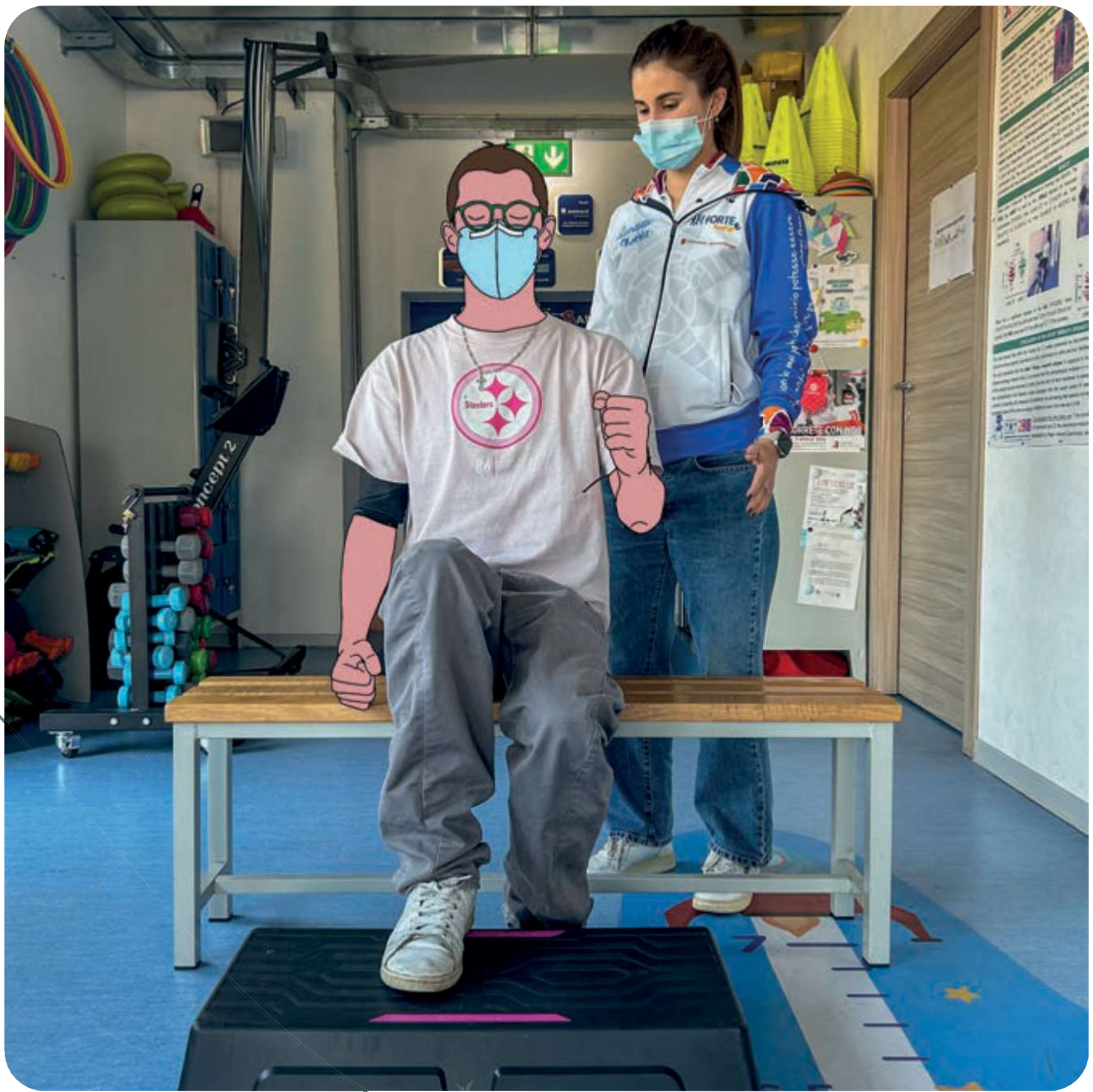

FROM SITTING POSITION

AVOID!

Rotating your torso.
Looking down.
Holding your breath.
